# Barriers to and facilitators of user engagement with web-based mental health interventions in young people: a systematic review

**DOI:** 10.1007/s00787-024-02386-x

**Published:** 2024-02-14

**Authors:** Thi Quynh Anh Ho, Long Khanh-Dao Le, Lidia Engel, Ngoc Le, Glenn Melvin, Ha N. D. Le, Cathrine Mihalopoulos

**Affiliations:** 1 Deakin Health Economics, School of Health and Social Development, Institute for Health Transformation, Deakin University, Melbourne, VIC Australia; 2 Monash University Health Economics Group, School of Public Health and Preventive Medicine, Monash University, Melbourne, VIC Australia; 3 School of Psychology, Deakin University, Melbourne, VIC Australia

**Keywords:** Digital mental health, Young people, Adolescents, User experience

## Abstract

Many young people (YP) are diagnosed with mental illnesses and require support. Web-based mental health interventions (W-MHIs) have been increasingly utilized by YP, healthcare providers, and parents due to reasons including convenience and anonymity. W-MHIs are effective in improving mental health in YP. However, real-world engagement with W-MHIs remains low. Therefore, understanding barriers/facilitators of user engagement with W-MHIs is necessary to promote W-MHIs and help users gain optimal benefits through higher engagement. This review aims to identify barriers/facilitators of user engagement with W-MHIs in YP aged 10–24 years. A systematic search of five databases for English language, peer-reviewed publications was conducted between January 2010 and February 2023. Studies examining factors influencing user engagement with W-MHIs, described as barriers or facilitators, were included. Study quality was assessed using the Mixed Methods Appraisal Tool. A narrative synthesis was performed. Of 4088 articles identified, 69 studies were included. Barriers/facilitators were reported by young people (63 studies), providers (17 studies), and parents/caregivers (8 studies). YP perceived that usefulness and connectedness were the most common facilitators, whereas low-perceived need was the most reported barrier. Both providers and parents reported that perceived usefulness for YP was the most common facilitator, whereas concerns about program effectiveness and privacy were noted as barriers. This review found that program- and individual-related factors were important determinants of engagement with W-MHIs. This review provides guidance on the future design and development of new interventions, narrowing the gap between existing W-MHIs and unmet needs of users.

**Supplementary Information:**

The online version contains supplementary material available at 10.1007/s00787-024-02386-x.

## Background and introduction

Globally, one in seven young people (YP) aged 10–24 has a mental disorder, accounting for 12% of the global disease burden at this age [1]. More than one in five of YP with a mental disorder experience severe mental illness [2]. Those suffering from a mental disorder at this age are at higher risk of having a disorder ten years later [3]. Nearly 50% of mental disorders have an onset before the age of 14, and 75% before 24 [4, 5], making adolescence and young adulthood a critical time to intervene and promote mental wellbeing. However, several barriers impede YP in accessing professional mental health support, including financial costs, social stigma, negative beliefs about mental health services, and low mental health literacy [6, 7]. Online mental health interventions, including web-based mental health interventions (W-MHIs) (i.e., those delivered on a website platform) and apps, can potentially overcome some access issues. Yet, online interventions may be inappropriate for individuals with severe mental illness due to their severe symptoms and limited cognitive and mental capacity [8].

Online interventions have been increasingly utilized, especially during the COVID-19 pandemic, largely due to their accessibility and anonymity. Zhou et al. found that 78% of online mental health interventions for youth aged 15–24 were delivered on web-based self-help platforms [9]. W-MHIs, especially web-based cognitive behavioral therapy (CBT), were found to be effective in managing common mental health conditions (e.g., anxiety, depression) in YP aged 10–24 [9, 10]. Meanwhile, limited evidence on effectiveness was reported in mental health apps, with only 3% of 293 apps having published research evidence [11]. That suggests W-MHIs offer a promising approach to expand mental health care for individuals, particularly those with mild and moderate symptoms.

User engagement with W-MHIs has been more commonly examined than mental health apps [12, 13]. Engagement refers to a dynamic process, which starts with a trigger (e.g., recommendation by health professionals), uptake of the program, that is followed by either a sustained engagement or disengagement [14]. Engagement can be assessed by objective measures (e.g., usage pattern) and subjective measures (e.g., user experience) [15]. Amagai et al. identified interchangeable terms were used for *user engagement*, such as *retention, adherence, compliance, completion, *etc*.* [16]. Greater engagement with W-MHIs can lead to mental health improvements [17]. Nevertheless, engagement remained low, with only 30% of YP completing at least three of 10 sessions in a web-based CBT program for anxiety [18]. Higher level of engagement were generally observed in web-based applications compared to web-based self-help platforms [9]. It is noteworthy that the reported engagement of unguided W-MHIs in trial-based research was roughly 1.1–4.1 times higher than real-world engagement of the same program [19]. Low engagement can be associated with the early dropout in the treatment, creating challenges in translating potential benefits of W-MHIs to the real world [14].

Engagement with W-MHIs can be influenced by a wide range of factors, including personal motivation, personal life, and quality of the program [20]. Based on qualitative data, Garrido et al. indicated that intervention-related factors (e.g., program content and technical glitches) could influence YP’s engagement with W-MHIs and other online interventions for anxiety and depression [21]. Liverpool et al. highlighted intervention and individual characteristics were barriers/facilitators of engagement with these interventions in children and YP [12]. However, most studies included in this review were conducted in the development and testing stages, revealing the gap in understanding factors influencing YP’s engagement in the real-world setting. Another review in 2021 focused on various types of online mental health interventions in adults (16 years and above), which did not report separate findings for YP [13]. Differences in attitudes towards mental health care between YP and adults, due to different lifestyles, preferences, and needs, suggest limited applicability of this review’s findings to YP [22, 23].

In addition to YP, healthcare providers (e.g., health professionals, program moderators) and parents are also end-users of W-MHIs. They may provide support to YP or actively participate in the program. Parents can motivate their children, especially those below 16 years, to engage with W-MHIs through their reminders or support [24]. Providers’ engagement can influence YP’s engagement with W-MHIs [25]. Absence of therapist participation in the W-MHI made it challenging in ensuring adolescents with depressive symptoms engaged in online CBT [26]. Yet, providers’ engagement can be impacted by a lack of established training and guidance [26].

To date, there are very few reviews investigating barriers/facilitators of YP’s engagement with W-MHIs [12, 21] and providers’ delivering W-MHIs [26], and no reviews about factors influencing parents’ engagement with W-MHIs for YP. Given the need to improve engagement with W-MHIs, particularly in YP (defined as aged between 10 and 24 [27]) beyond the research setting, this review aims to examine barriers and facilitators of engagement with W-MHIs for YP from the perspectives of YP, healthcare providers, and parents.

## Method

This review was prospectively registered on PROSPERO (CRD42022290298) and conducted in compliance with the Preferred Reposting Items for Systematic Reviews and Meta-Analyses (PRISMA) guidelines [28].

### Search strategy

A literature search was conducted in MEDLINE, PsycINFO, CINAHL Complete, Global Health, and Embase. Search terms for titles and abstracts included four concepts: (1) young people, (2) web-based interventions, (3) mental health, and (4) barriers or facilitators (see Online Resource 1 for details of the search strategy).

### Inclusion and exclusion criteria

An article was included if it reported the use of a W-MHI targeting YP aged 10–24 years (as defined by the United Nations [27]) and investigated users’ barriers/facilitators of engagement with the intervention. Our primary focus was on the age range of 10–24 years; however, for studies where a minority of participants were above 24 or below 10, we decided to include these studies in our systematic review if the age range was reported and mean age was between 10 and 24 years. The included articles were empirical studies, peer-reviewed, written in English, and published from January 2010 to February 2023. Online health platforms, including web-based platforms, have rapidly evolved and there might be increasing new evidence-based tools that promote mental health care [29]. To focus on the current state of W-MHIs and avoid discussing potential out-of-date interventions, we included publications from 2010 onwards as this time frame has been applied in previous systematic reviews of online mental health interventions [13, 30].

In this study, W-MHIs are those delivered via web-based platforms (e.g., a website, a social media platform, or emails), and used for mental health support, prevention, and treatment. Interventions with the sole purpose of mental health assessment were excluded. Computerized interventions were excluded if delivered via a software computer program without an online network, or if they were delivered via telephone using interactive voice responses (e.g., telehealth). W-MHIs can encompass components delivered via an app or a face-to-face session. Barriers to W-MHIs are factors that might discourage or hinder users from engaging with W-MHIs. This might include reasons for disengagement and factors that users dislike. Conversely, facilitators of W-MHIs are factors that users rated as important or facilitated them to use these programs.

Articles were excluded if they were published in a language other than English and reported an intervention that was not delivered on the web. Non-English publications were excluded due to limited resources for translation services [31]. We also excluded pilot studies and studies reporting interventions that were in the development process, targeted substance use, wellbeing, and mental health risk factors (e.g., bullying), and primarily aimed at individuals beyond 10–24 years. Studies reporting interventions for providers, or parents of YP were also excluded.

### Study selection and extraction

**Fig. 1:**
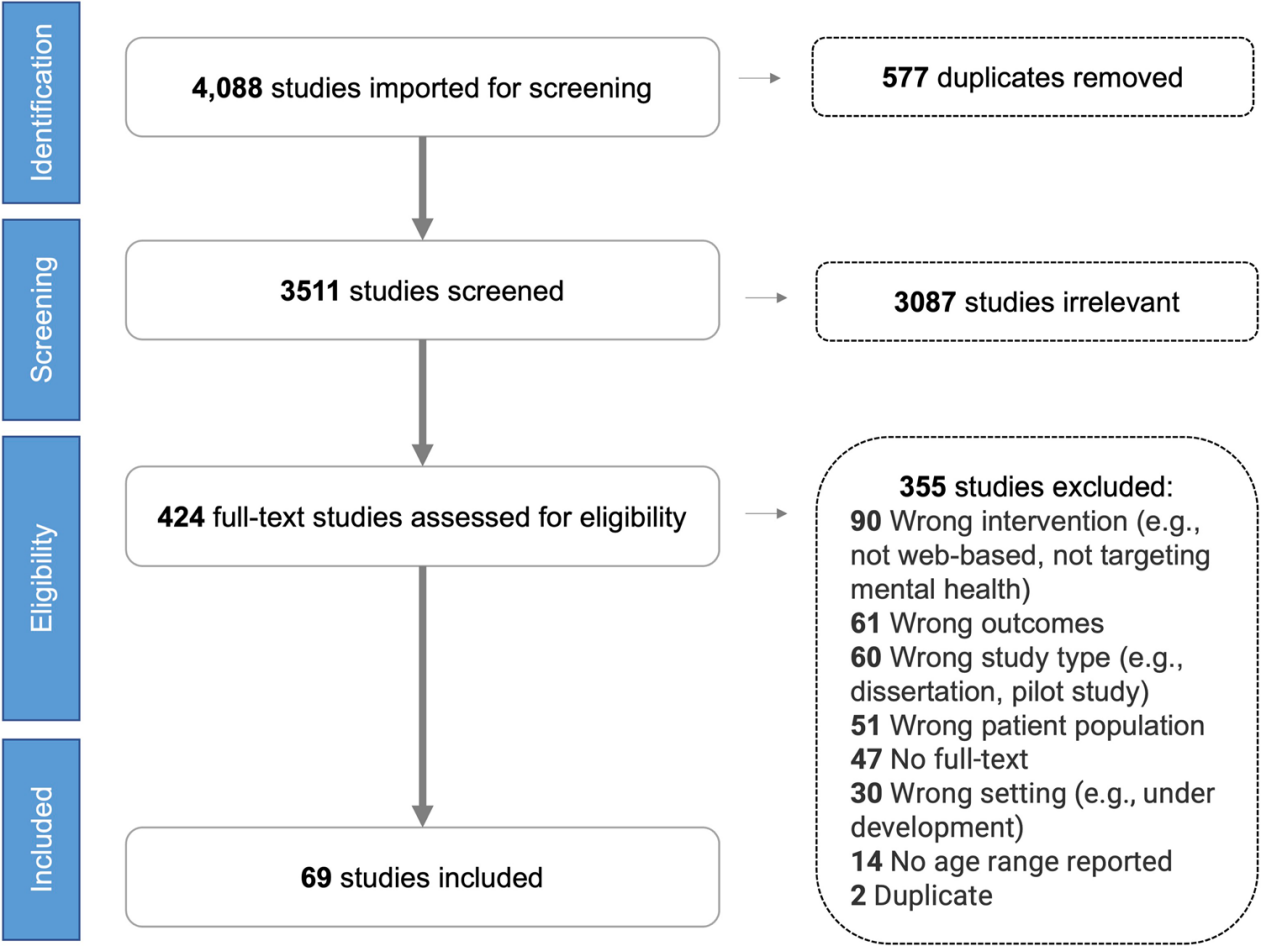
PRISMA flow diagram of article screening and inclusion

The articles identified were uploaded to Covidence (www.covidence.org). After removing all duplicates, two independent reviewers (TH and NL) screened the remaining articles for eligibility in two stages, including title and abstract, and full-text screening. A pilot screening was conducted with the first 100 articles in each stage to check the agreement between two reviewers. There was fair to moderate agreement between the two reviewers (Cohen’s Kappa value = 0.30 and 0.42 for title and abstract, and full-text screening, respectively). It is suggested that a score as low as 0.41 in health-related studies might be acceptable [32]. Any discrepancies were resolved with the third reviewer (LL). The decision of the third reviewer was final. Figure 1 illustrates a PRISMA flow diagram of the screening articles. Findings of all included studies, defined as ‘all of the text labeled as ‘results’ or ‘findings’ in study reports’ [33], were extracted to an Excel spreadsheet for subsequent data synthesis.

### Quality assessment

Given the heterogeneity of study methods (i.e., qualitative, randomized-control trial (RCT), uncontrolled trial, descriptive, and mixed methods), the Mixed Methods Appraisal Tool (MMAT) version 2018 was used to critically appraise the study quality [34]. The reliability and efficiency of the MMAT was evaluated in several studies [34–37]. The MMAT contains two screening questions and five follow-up questions to appraise methodological quality depending on the study methods. As this review included empirical studies only, we decided not to use the two screening questions [34]. Five criteria in the chosen category were rated (“Yes”, “No”, “Can’t tell”). The quality score was calculated by summing the number of ‘yes’ items [38]. On a range from zero to five, study quality was categorized into ‘low’ (score of two or below), ‘moderate’ (score of three), and ‘high’ (score of four or five). All studies were included regardless of their MMAT score. A sensitivity analysis was performed to examine the impact of low-quality studies on the findings, whereby low-quality studies were removed and the subsequent impact on results and conclusions was examined. Two reviewers (TH and NL) independently carried out the quality assessment. Any discrepancies were discussed between the two reviewers and consulted with the third reviewer (LL).

### Data synthesis

This review took an epistemological perspective of constructivism, that allowed us to grasp and translate meaning of facts (existing data) and constructed knowledge (themes/subthemes) [39]. We adopted a narrative synthesis approach to data synthesis [40]. Results were synthesized separately for qualitative and quantitative components, using thematic analysis [41]. For qualitative results, each barrier/facilitator of user engagement was assigned a code, and we reorganized the data according to these codes (e.g., ‘increase mental health knowledge, ‘learn about coping skills’). We then refined codes through an iteration process and grouped codes into appropriate subthemes (e.g., ‘perceived usefulness’). For quantitative results, factors scored favorably (or unfavorably) were considered facilitators (or barriers). Factors with less than 10% of participants endorsed were not included to develop subthemes but instead were taken into consideration during the subsequent sensitivity analysis. We categorized each barrier/facilitator into an appropriate existing qualitative subtheme and developed a new subtheme if it did not fit in any existing subtheme. Once we developed subthemes for both qualitative and quantitative findings, we observed the similarities among these subthemes, and collated them into themes (e.g., ‘intervention-related factor’).

## Results

### Study characteristics

Out of 4088 studies, 69 studies were included in the review (Fig. 1), with 31 quantitative, 23 qualitative, and 15 mixed-methods studies.

Study characteristics are described in Online Resource 2. Fifty-nine studies (86%) were conducted in high-income countries, including Australia and New Zealand (*n* = 20, 29%), the US and Canada (*n* = 14, 20%), UK (*n* = 8, 12%), and Sweden (*n* = 5, 7%). Sample sizes varied widely: ranging from 4 to 118 in qualitative and from 14 to 7849 in quantitative components. Barriers/facilitators in using W-MHIs were reported from YP (*n* = 63, 91%), healthcare providers (*n* = 17, 25%), and parents (*n* = 8, 12%). Of 63 studies reporting YP’s perspectives, 27 studies (46%) targeted adolescents under 19 years, 21 (36%) targeted YP aged between 16 and 25 years, and the remaining targeted cohorts aged 10–25 years. Four studies targeted minority groups, including black young men [42], LGBTIQA + people [43, 44], and YP of a refugee background [45]. The study population was predominantly young women (*n* = 54, 86%). Barriers/facilitators were captured either qualitatively (i.e., interviews (41%), focus groups (13%)), or quantitatively (i.e., self-reported surveys using rating scale (57%) or open-ended questions (9%), or observational methods (3%)).

### Intervention characteristics

Of the 69 included studies, 58 studies (84%) reported participants’ feedback of using a specific intervention and the remaining studies explored general W-MHIs without focusing on a specific program. Intervention characteristics are presented in Online Resource 3.

Forty-two studies (61%) reported interventions targeting anxiety, depression, and/or stress, three (4%) targeted obsessive–compulsive disorder, three (4%) targeted suicidal ideation, and two (3%) targeted eating disorders. The remaining (*n* = 19, 28%) targeted mental wellbeing without a specific condition. The included interventions were primarily delivered via a website only (*n* = 61, 88%). Other platforms included web-apps (e.g., delivered via a website and an app) (*n* = 6, 9%), and a combination of web-based and face-to-face sessions (*n* = 2, 3%). The majority of W-MHIs were based on CBT (*n* = 37, 54%). Other approaches included psychoeducation (*n* = 4, 6%), social networking (*n* = 2, 3%), positive psychology (*n* = 2, 3%), and a combination of different approaches (*n* = 10, 14%). The remaining studies did not report the intervention approach.

Overall, 39 studies reported guided W-MHIs, 16 reported unguided W-MHIs, and 14 did not specify. The guidance was provided by health professionals (e.g., therapists, clinicians, and physicians) or non-health professionals (e.g., school staffs), reported in 24 and 13 studies, respectively. Peer support was embedded in four interventions. Intervention duration varied, ranging from single to 40 sessions and each session lasted between 5 and 60 min.

Due to the great variance in study methodology, study population and interventions, a meta-analysis was infeasible.

### Barrier/facilitator themes

Barriers/facilitators from YP’s, healthcare providers’, and parents’ perspectives are summarized below. Barriers/facilitators identified in each study are provided in Online Resource 4.

### Young people’s barriers and facilitators of engagement with W-MHIs

**Fig. 2:**
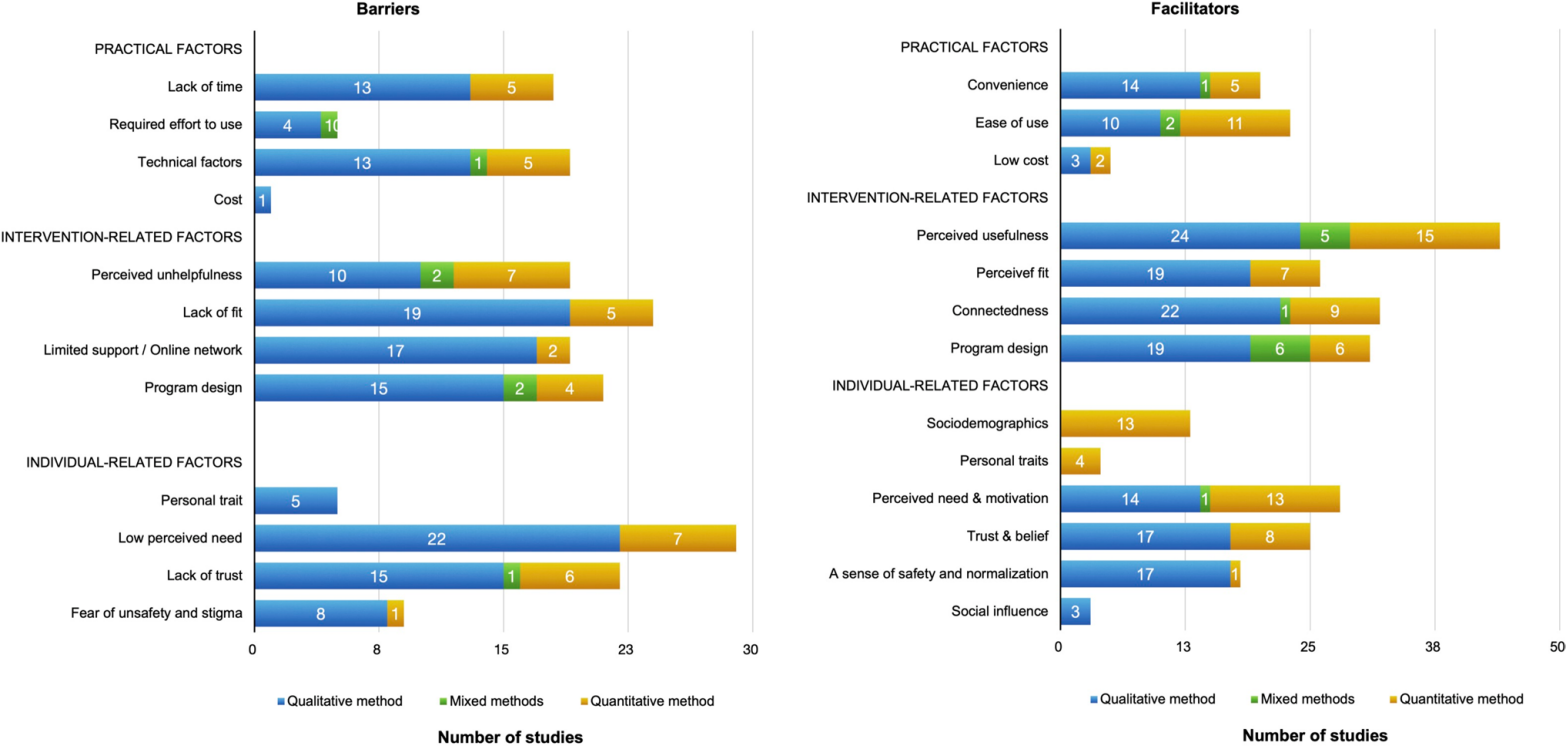
Young people’s barriers and facilitators of engagement (*n* = 63 studies)

There were 63 studies reporting YP’s perspectives regarding their barriers/facilitators of engagement with W-MHIs. Three overarching themes were developed, including practical factors, intervention-specific factors, and individual-specific factors. There was some overlap among themes due to the influence of factors associated with user’s perception and behavior to use. For instance, user-specific factors (e.g., perceived need and interest) might drive user’s perceived program usefulness. Intervention-specific factors (e.g., program features and usefulness) might impact user interest in W-MHIs, consequently influencing user engagement. Barriers/facilitators were reported more frequently in qualitative studies than quantitative studies, with the exception of sociodemographic variables. A summary of subthemes and their distributions across all studies are outlined in Fig. 2.

### Theme 1: practical factors

#### Convenience but lack of time

Twenty (32%) studies found that YP’s engagement with W-MHIs was facilitated due to the accessibility that allowed them to access it anywhere and at any time [42, 46–62] and the ease of incorporating their use into YP’s schedule [50, 57, 58, 63]. This facilitator was endorsed by 59–95% of YP [46, 49, 51, 52, 56]. In contrast, some YP preferred to complete sessions on schedule [54]. Lack of time or busyness was a barrier (*n* = 18, 29%) [45, 46, 48, 51, 53, 54, 56, 57, 62, 64–73], endorsed by 42–76% of participants [56, 65, 67, 69].

#### Ease of use and technical factors

Being easy to use was a facilitator of engagement (*n* = 23, 37%) [43–46, 54, 57, 58, 63, 74–81], endorsed by 63–90% of study participants [46, 52, 56, 68, 69, 74, 82–84]. Disengagement could be caused by technical factors (*n* = 20, 32%), primarily related to connection issues [43, 46, 55, 57, 68, 73, 79] and if the program was not working properly [54, 58, 62, 79, 85].

#### Cost

Low cost of access was identified as a facilitator in five studies [56, 57, 59, 60, 78]. Despite the relatively low cost compared to in-person therapies, cost of W-MHIs was reported as a barrier in one study [59].

### Theme 2: intervention-specific factors

#### Perceived usefulness or unhelpfulness

Perceived usefulness of W-MHIs was the most frequent intervention-related facilitator of engagement (*n* = 44, 70%). Perceived unhelpfulness was a barrier to YP’s engagement (*n* = 18, 29%). Program usefulness was assessed quantitatively in 20 studies. There were mixed views: 27–88% of participants perceived the program as useful [56, 60, 71, 74, 86–89], whereas 12–38% found it unhelpful [64, 65, 86, 88, 89]. Perceived usefulness referred to immediate benefits [42, 53, 76] such as symptom relief, mood improvement [44, 48, 77, 90, 91], mental health education [42, 44, 48–51, 53–55, 57, 58, 62, 63, 66, 68, 70, 71, 78, 79, 82, 85, 90–93], and self-reflection [42, 46, 48–50, 55, 66]. The ability to disclose one’s feelings rather than avoiding them was endorsed by 69–73% of study participants [49]. However, some found it difficult to portray feelings in writing [46, 47, 94]. In addition, informative content was a facilitator of engagement [45, 46, 60, 64, 75, 80, 81], while content that was already known [55, 70, 75, 82, 90], repetitive [54, 55, 68, 79], too simple [46] or unspecific [56, 75] was a barrier.

#### Perceived fit or lack of fit

An appropriate program was a facilitator (*n* = 26, 41%), whereas a lack of fit was a barrier to YP’s engagement (*n* = 25, 40%). YP’s engagement was enhanced if W-MHIs were age-appropriate [55, 62, 79] and relevant [44, 45, 48–50, 53, 58, 62, 76, 82, 85, 90, 95]. The lack of age-appropriateness [46, 53, 55, 62, 68, 78, 79] and irrelevance (e.g., cultural difference [45], difficult tasks [46, 54, 56–58, 87], lengthy modules [58, 60, 62, 72, 76, 90]) may result in poor engagement. It is noteworthy that YP had different views about module duration and module difficulty depending on the types of intervention. For example, users of a web-based anxiety program preferred a 10–15 min module [76] while users preferred a longer session (more than 30 min) in a web-chat counseling service [96]. When asked about W-MHIs in general, YP were reluctant to spend more than 30 min at a time [78]. YP’s engagement was impeded if it required significant time and effort for program completion [45, 48, 55, 72]. It is noted that this barrier was only found in W-MHIs with CBT components. Four studies reported YP’s views about moderators’ approach. An appropriate approach from moderators (e.g., emotional support) was a facilitator [97], whereas an excessively enthusiastic approach could make YP feel artificial [98] and might not be suitable for YP with depression [46]. Excessive moderation creating an atmosphere of control was another barrier to YP’s engagement [90]. Perceived fit of W-MHIs was enhanced by the personalization of W-MHIs [51, 54, 75, 90, 93], which provided individualized interaction and feedback [48, 51, 90, 98]). A lack of individualization was a barrier [53, 62, 85, 93].

#### Connectedness and nature of online network

Connectedness, referring to the ability to connect with others, was the second most common facilitator of engagement (*n* = 32, 51%). The impersonal nature of an online network and a lack of support were the second most common barriers to engagement (*n* = 19, 30%). YP valued human contact [57, 93, 99], especially with those having similar lived experience [44, 47, 53, 54, 66, 79, 90, 92, 98]. Although 75–84% of YP recognized the importance of human contact, particularly with professionals, 55–77% were reluctant to use online interactions (e.g., online chat or discussion) during their difficult times [99]. The online network could be a barrier as YP were unable to connect closely with other users [90, 98] and could be negatively impacted by other users (e.g., through jokes [57] or being ignored in the online platform [98]).

Connectedness helped YP feel supported [42, 53, 64, 66, 75, 80, 82, 88, 97]. Health professionals (e.g., therapists) were the most common source of support in W-MHIs [42, 46, 50, 53, 55, 60, 63, 66, 82, 85, 88, 90, 97, 98, 100]. A lack of therapist support (e.g., being unable to ask questions) was a barrier in using W-MHIs, reported by 62% of study participants [56]. Besides, peer support [64, 66, 80, 90, 98] and parental support [50] were likely to enhance YP’s engagement. By being involved in the program, parents could better understand their children’s symptoms and encourage them to engage in the program. Engagement was further improved if the support was persistent [54], instantaneous [46, 50, 55, 59, 85, 90, 98], and easy to access (e.g., via email [54, 79] or chat [68]). The lack of timely responsiveness [46, 54, 55], insufficient, and infrequent support [45, 53, 55, 56, 61, 70, 79, 85, 90] were barriers to engagement. There were mixed views about the impersonal nature of online support. Some preferred to talk online and therefore, considered it as a facilitator [43, 57, 77, 90, 92, 94]. Some preferred contact with therapists via videoconferencing rather than messaging [45]. The impersonality of W-MHIs might be a barrier, leaving YP feeling disconnected [43, 54, 55, 57, 91, 94] and challenged in building trust and rapport with health providers online [57, 92, 94].

#### Program design

Program design (e.g., features, layout, structure, etc.) could influence YP’s engagement in W-MHIs and was reported in 36 studies (31 reporting facilitators and 21 reporting barriers).

YP’s engagement was facilitated by an attractive design [46, 57, 64, 75, 79, 90] and greater interactivity (e.g., engaging quizzes, gamification) [42, 55–57, 62, 90, 99]. Lacking interactivity and entertainment resulted in high disengagement with W-MHIs [44, 66, 68, 70, 85]. The utilization of informal means of communication (e.g., chat, messages) [43, 77] and combined text, videos, and pictures [51, 53, 58, 63, 75, 95, 99] facilitated W-MHI engagement. However, too much text [46, 48, 55, 62, 68, 75, 90, 92] or pictures [46, 54] were reported as barriers. It is noteworthy that YP with depression disliked a design representing excessive happiness [46]. Moreover, favorable features (e.g., reminders [46, 62, 75], progress tracking [54, 56, 85], rewards such as a treasure chest [55]), tended to increase W-MHI engagement. In contrast, unfavorable features lowered YP’s engagement (e.g., black and white design [58]). Furthermore, YP’s engagement was improved when W-MHIs were logically structured [46, 62, 75, 90] and easy to navigate [46, 58, 59, 75, 92, 101], and language used was informal [55, 77], easy-to-understand [45, 63, 75, 80, 82, 85], non-pathologizing, [92] and positive[57]. The lack of responsive text alignment and navigation remained barriers in web-apps [57, 76].

### Theme 3: individual-specific factors

#### Sociodemographic factors

Sociodemographic variables (e.g., age, gender, family background) impacted user engagement with W-MHIs (*n* = 13, 21%). Males and females had different perspectives towards the utilization of W-MHIs [88, 89]. For instance, males were more likely to seek intrapersonal advice on web-based mental health forums, whereas females tended to seek interpersonal support [76]. Higher engagement was found in younger ages [71, 80, 95, 102], females [52, 69, 82, 84, 89, 103], those with higher education [67, 82], living with family [88] and having more educated parents [69].

#### Personal traits

Personal traits refer to YP’s characteristics that impact their thoughts, feelings, and behaviors. Personal traits were described as barriers or facilitators of engagement, reported by YP using CBT-based programs or those being asked about their attitudes towards W-MHIs in general (without focusing on a specific program). However, such results were not found among YP using social networking programs. A lack of confidence [45, 51, 76] and determination [55, 57] impeded YP from using W-MHIs. Likewise, quantitative findings found that self-reliance [104], self-esteem [102], and a greater autonomy [46, 48, 50, 51, 57, 79, 103] tended to increase usage activity. Embarrassment had varied influence on the engagement. While W-MHIs could alleviate embarrassment associated with face-to-face services [56, 104], shy people faced challenges in online social interaction [55, 90].

#### Attitudinal factors

Attitudinal factors comprise attitudes, beliefs, and norms that influence an individual’s perception and actual behavior [105] to engage with W-MHIs and were reported in 56 studies (89%).

##### Perceived need or low-perceived need

Perceived need to use W-MHIs was a facilitator (*n* = 29, 46%), driven by factors such as acceptance of W-MHIs [43, 46, 48, 53, 54, 57, 75, 78], a preference for online services [56, 104], and high motivation [50, 58, 79, 94, 104]. YP’s motivation was influenced by awareness of mental health condition [69, 75, 90, 102, 104], curiosity about W-MHIs [60, 62], or enjoyment derived from W-MHIs [56, 63, 64, 66, 68, 80, 84, 85, 90, 101].

In contrast, a low-perceived need and a lack of motivation were barriers to YP’s engagement (*n* = 28, 44%). Mental illness symptoms (e.g., hopelessness, low energy) [45, 46, 91], insufficient understanding of mental health and available services [23, 43, 46, 47, 54, 58, 59, 64, 76–78, 96], and a need for different help [50, 73, 104] (e.g., talking to someone directly [62]) might weaken their need and interest in using W-MHIs.

##### Trust or concern about the program and privacy

Eighteen studies (29%) reported that beliefs in anonymity, privacy, and confidentiality were facilitators [42, 45, 48, 54, 57–60, 62, 66, 77, 79, 88, 90, 92, 94, 95, 99, 106], especially for those not wishing to visit a health professional [57]. Trust in the program credibility [46, 57, 59, 82, 88, 95, 99, 100] and effectiveness [50, 52, 54, 76] (*n* = 10, 16%) as well as the ability to build trust with others online [50] could enhance engagement. Nevertheless, concerns about privacy and confidentiality (*n* = 14, 22%, [46, 47, 50, 56–59, 65–67, 69, 76–79]) (e.g., leaking personal information [99]) and uncertainty about the potential effectiveness of W-MHIs (*n* = 11, 17% [43, 45, 47, 51, 54, 57, 67, 91, 94, 104]) remained important barriers.

##### Feeling safe and fear of stigma

Feeling safe (e.g., feeling secure [46, 50, 79] and not being judged [42, 53, 58, 77, 90, 98]) could enhance YP’s engagement with W-MHIs, whereas feelings of insecurity during the online mediation sessions [87] and anxiety stemming from past experience could impede user engagement [94]. Engagement was improved if YP could overcome stigma and normalize their mental health problems [47, 48, 50, 53–55, 57, 59, 70, 71, 75, 90]. However, stigma remained a barrier to W-MHI engagement [43, 57, 76, 104], especially in LGBTIQA + young adults [43]. Moreover, fear of potential negative impacts on their problems [47, 54, 75, 92, 98] and causing harm to others [98] were barriers to W-MHI engagement. It is noteworthy that feeling safe was endorsed by users of a specific intervention. In contrast, stigma was primarily reported by YP over 18 years who were asked about W-MHIs in general rather than a specific intervention.

##### Social influence

Social norms influenced YP’s engagement with W-MHIs [52, 78, 79]. Social factors could impede YP’s engagement, such as insufficient active users [54, 62, 64, 90, 98], being ignored by others [57, 66, 98], and not knowing others on the online social network [90]. The impact of social influence was reported in qualitative studies only.

### Healthcare providers’ perspective on the barriers and facilitators to mental health service provision

**Fig. 3:**
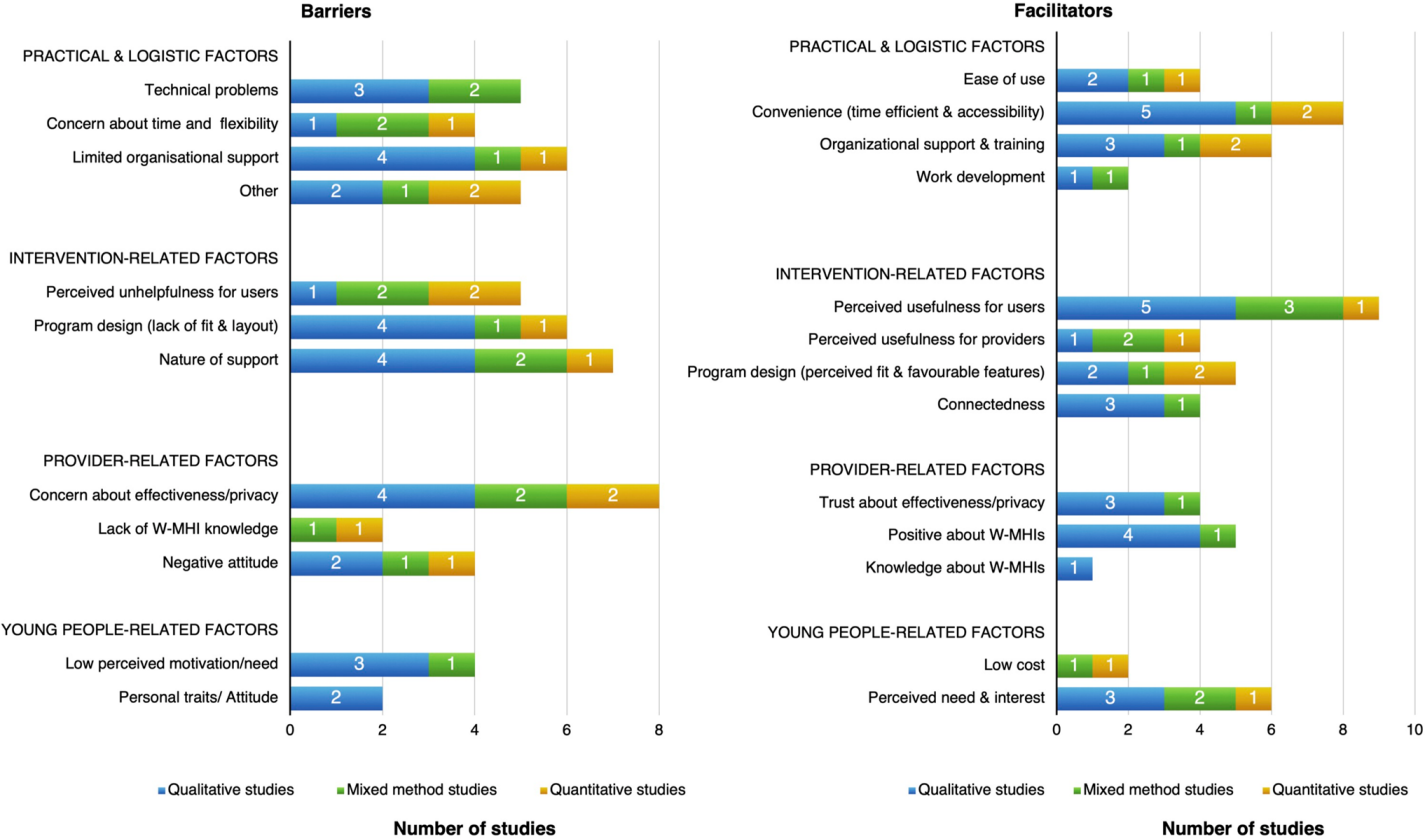
Healthcare providers’ barriers and facilitators of W-MHI provision (*n* = 17 studies)

Healthcare providers can be involved in the provision of W-MHIs by offering guidance and support to YP. Seventeen studies reported providers’ perspectives about their barriers/facilitators of W-MHI provision for YP. Four overarching themes were identified, including practical and logistic factors (*n* = 12, 71%), intervention-related factors (*n* = 8, 73%), provider-related factors (*n* = 5, 46%), and YP-related factors (*n* = 6, 55%). Barriers and facilitators were reported more commonly in qualitative studies than quantitative and mixed methods. Subthemes and their distributions across study methods are outlined in Fig. 3.

### Theme 1: practical and logistic factors

The provision of support in W-MHIs was enhanced by the flexibility to deliver W-MHIs at anytime and anywhere (*n* = 8, 47%) and ease of use (*n* = 4, 24%); whereas technical issues could hinder the seamless delivery of support [57, 75, 79, 97, 107].

Logistic factors, mostly organizational, were reported in nine studies (53%). Helpful training and adequate organization support enhanced providers’ confidence and facilitated the provision of support to YP [61, 97, 107–110]. The potential expansion of mental health services to underserved groups was another facilitator of W-MHI provision [57, 61]. The integration of W-MHIs in healthcare was perceived as a facilitator due to the pre-determined treatment program structure, which lessened the cognitive burden in session preparation [24]. However, it was a barrier if providers faced an increasing workload [110]. Limited resources and a lack of training were common barriers [43, 57, 79, 107, 109, 110]. In the school setting, school counselors and staffs reported additional barriers, including time constraints in the school schedule and program incompatibility with school values [109].

### Theme 2: intervention-related factors

Facilitators of providing W-MHIs included perceived program usefulness for YP and providers (*n* = 11, 61%), connectedness (*n* = 5, 28%), and favorable program design (*n* = 5, 28%) (e.g., individualization [24], and parent’s involvement [108]). Barriers to W-MHI provision included the nature of online platform (e.g., asynchronous communication) (*n* = 6, 33%), perceived risk or unhelpfulness for YP (*n* = 5, 28%), and a lack of fit (*n* = 3, 17%).

Consistent with YP’s perspectives, providers valued the ability of YP to connect with others and receive professional support in W-MHIs [43, 57, 108]. The ‘faceless contact’ was reported as a facilitator to YP’s engagement [94, 107] but it remained an important barrier to conveying information and building therapist rapport [24, 57, 61, 94, 107]. A standardized structure was a facilitator as it provided *‘equal care’* to everyone, though it was a barrier due to a lack of individualization for YP [24] and the potential of feeling boredom by providers [61].

### Theme 3: provider-related factors

Providers were more likely to recommend the use of W-MHIs if they held positive attitudes about W-MHIs (*n* = 5, 28%). This included finding W-MHIs acceptable [57, 111], trusting in program effectiveness and privacy (*n* = 4, 22%) [24, 43, 57, 79], and perceiving their role as intermediaries between the intervention and service users (e.g., providing support to users) [24, 79]. On the other hand, a resistance to change [110] and uncertainty about user privacy [57, 79, 109, 110] and program effectiveness [57, 61, 107, 111] impacted W-MHI provision. In addition, the lack of mental health knowledge [109] and experience with online programs [57, 61] were barriers, as providers lacked confidence in delivering support online.

### Theme 4: young people-related factors

As YP were the primary users of W-MHIs, their engagement played an important role in the provision of W-MHIs (*n* = 8, 47%). Providers emphasized that YP’s engagement could be enhanced if YP perceived a need to use W-MHIs when being at risk of mental illness [57, 109] and facing difficulties with in-person services [24, 57]. In contrast, providers highlighted a lack of motivation [94] and the potential for procrastination to postpone the treatment could lead to poor engagement among YP [24].

### Parents’ perspective on barriers and facilitators to their children’s and their engagement with W-MHIs

**Fig. 4:**
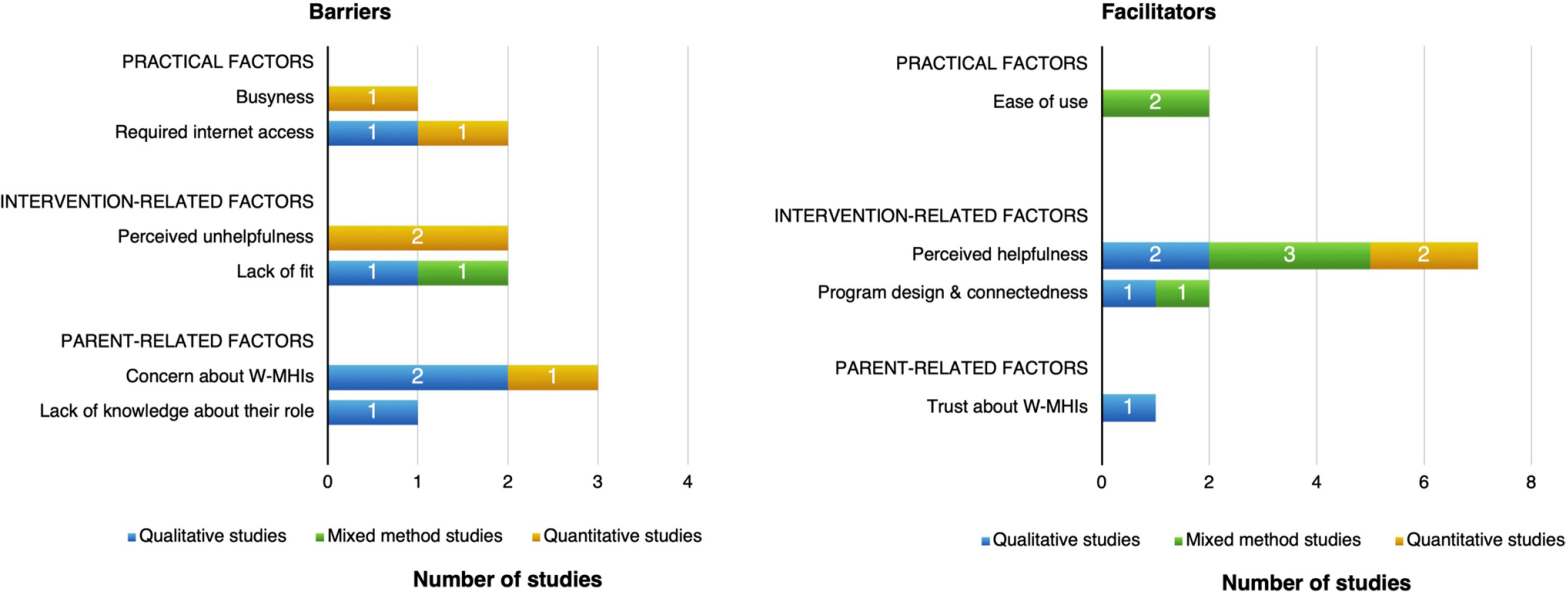
Parents’ barriers and facilitators of engaging with W-MHIs (*n* = 8 studies)

Eight studies (two qualitative, three quantitative, and three mixed-method) reported parents’ perspectives about their barriers/facilitators and their children’s barriers/facilitators of W-MHI engagement. Of these, five examined W-MHIs that incorporated parental components [63, 65, 75, 86, 93]. Three overarching themes were identified, including practical factors (*n* = 3, 38%), intervention-related factors (*n* = 8, 100%), and parent-related factors (*n* = 3, 38%). Barriers were reported across study designs, whereas facilitators were primarily identified in qualitative and mixed-method studies. Subthemes and their distributions by study designs are outlined in Fig. 4.

The most common facilitator of parents’ and their children’s engagement with W-MHIs was perceived helpfulness of W-MHIs (*n* = 7, 88%, [51, 63, 71, 75, 78, 86, 93]). The most common barrier to their engagement was concern about the program (*n* = 3, 38%) (e.g., program effectiveness [51], privacy [65], risk of internet addiction in YP [78]). Other barriers included required internet access [65, 78] and a lack of fit (e.g., irrelevance [65], a lack of interactivity [93]).

### Quality assessment

Forty-four (64%) studies were rated as ‘high’ quality, 19 (28%) ‘moderate’ and 6 (9%) ‘low’. All qualitative and non-RCT studies were ‘high quality’. For RCT studies, outcome assessors were unblinded to the intervention, as it was unavoidable due to the nature of intervention. The weakness of descriptive studies was largely related to non-representative samples. The majority of mixed-method studies lacked a rationale for using both qualitative and quantitative methods and were rates as moderate quality in the quantitative component. The quality assessment for all included studies is presented in Online Resource 5.

### Robustness of data synthesis

We conducted a sensitivity analysis by excluding six ‘low quality’ studies [56, 62, 64, 67, 73, 84]. There was no change in subthemes and minimal change in the percentages across all subthemes. The most evident change was observed in the lack of perceived need, which became less predominant but remained the most common barrier. In conclusion, the overall results remained unchanged.

## Discussion

### Overview of principal findings

This review provided a comprehensive synthesis of YPs’, healthcare providers’ and parents’ perspectives about barriers/facilitators of their engagement with W-MHIs. All users agreed that the most common facilitator was the perceived usefulness of W-MHIs for YP. The perception that W-MHIs could help YP improve their mental health knowledge and well-being would enhance YP’s and their parents’ engagement with W-MHIs and facilitate the service provision by providers. The most common barriers were YP’s low-perceived need to use W-MHIs and providers’ and parents’ concerns about program effectiveness and privacy for users. Similar barriers/facilitators and subtheme distributions were found in different types of interventions. Most barriers/facilitators of engagement were reported more frequently in qualitative than quantitative findings. Overall, our synthesized qualitative data was consistent with quantitative data.

### Interpretation of findings

Over half of the studies included in this review were published from 2020 onwards, which is likely attributable to the increased barriers in accessing face-to-face mental health services during the Covid-19 pandemic. The transition of most psychological interventions away from face-to-face format (at least temporarily), due to the pandemic restrictions, contributed to increased utilization of online mental health care during the pandemic [112]. With the observed growth in mental health problems, particularly among YP, during the pandemic [112], there was a greater research interest in the adoption and use of W-MHIs along with their facilitators and barriers to use. The majority of the included studies were conducted in high-income countries, reflecting a gap in the literature in this area from low- and middle-income countries (LMICs). Previous research on 49 countries representing all regions of the world found that the low access to mental health care was associated with low national income [113]. Less advanced technology development, limited resources [114], and paucity of research on the effectiveness and cost-effectiveness of these interventions in LMICs make it challenging to disseminate W-MHIs in these countries [74, 115–117]. This can partly explain why there are less studies examining user’s feedback on W-MHIs in LMICs. In addition, we noted that anxiety, stress, depression, and general mental wellbeing were the most common targets of interventions included in this review (93%). This is consistent with Kaonga and Morgan’s review [118], which identified 46 out of 61 online interventions targeting depression or general mental health and wellbeing. The high prevalence of anxiety and depression disorders globally [119] positions anxiety and depression as the primary targets for online mental health programs. Moreover, we found that the majority of W-MHIs included in our review were CBT-based. This can be explained by the increasing evidence of the effectiveness of online CBT-based interventions in improving mental health wellbeing [120], particularly in reducing anxiety and depression [121, 122].

The barriers/facilitators of user engagement identified in this review align with the extended Unified Theory of Acceptance and Use of Technology (UTAUT) [123]. According to this model, performance expectancy (e.g., perceived usefulness), effort expectancy (e.g., users’ comfort and acceptance in using a technology), social influence, perceived ease of use, and perceived enjoyment have positive impact on user’s continuance usage behavior, whereas perceived risk and cost indirectly impede the continuance usage behavior. In line with this model, our review found that perceived usefulness was the most common facilitator to user engagement. Perceived usefulness, and consequently user engagement was impacted by any perceived risk or doubt about program effectiveness and privacy. This finding is further supported by several studies about online health interventions. For instance, Horgan et al. reported that a lack of trust in websites was a barrier to young students accessing online services [124]. Borghouts et al.’s review also endorsed the important role of trustworthiness in driving user engagement with online mental health programs [13]. These concerns persist because W-MHIs have been seen to be effective only for some groups of population (e.g., those with mild or moderate symptoms [94]) and the therapeutic effect might not last long [61, 91]. Concerns about program effectiveness might also relate to the absence of face-to-face contact, that reduces the individualization [62] and causes ambiguity in communication [94]. We also found that perceived ease of use was another key influencing factor to user engagement. It may be enhanced by program design (e.g., user-friendly, easy to navigate), accessibility and familiarity to digital technology. Yet, several technical issues can put off the ease of use of W-MHIs.

From YP’s perspective, the connectedness embedded in W-MHIs, particularly with health professionals, emerged as the second most common facilitator. This aligns with previous studies, that identified therapist support as one of the leading facilitators of seeking mental health help [6] and thus, enhancing engagement with online mental health programs [125]. Moreover, the involvement of health professionals was found to be associated with improved treatment outcomes [126, 127], potentially enhancing YP’s perceived usefulness and engagement with W-MHIs. YP specifically appreciated the connectedness with those having similar lived experiences. This is because YP may have an empathic connection and perceive support from these people as more credible [90]. Moreover, our findings about intervention-related factors largely mirrors Garrido et al.’s review, that identified the features liked and disliked by YP in online mental health interventions [21]. However, we found that the most prominent barrier to YP’s engagement with W-MHIs was not related to intervention characteristics but individuals themselves. Low perceived need and motivation to use W-MHIs was found to be the most common barrier to YP’s engagement. This is in accordance with the extended UTAUT model, that states perceived enjoyment is a determinant of technology engagement [123]. Disinterest or low-perceived need to use W-MHIs might be attributed to YP’s health condition (e.g., low mood [91, 94]), insufficient mental health knowledge [76], a preference for different help [62, 67, 73], and a lack of fit of W-MHIs.

Our findings align with Liverpool et al.’s review [12], revealing both intervention-specific and person-specific factors influencing YP’s engagement. In addition, we identified influential factors such as a sense of normalization, social influence, and the absence of face-to-face contact. However, in contrast to Liverpool et al., we found diverse perspectives about the use of rewards depending on age and type of rewards. Adolescents found the use of a treasure chest as a reward motivating, whereas YP over 18 years perceived it as childish [55]. YP appreciated verbal rewards made by moderators [97]. In our review, mixed views were also found about the program’s age-appropriateness irrespective of whether users were young adolescents or older. This can be attributed to the development in cognitive, psychosocial and emotional capacity during adolescence to adulthood that can variably affect perceptions and emotions [128]. Our review advanced the current literature by examining barriers/facilitators of engagement with W-MHIs from broad perspectives of healthcare providers, YP and their parents.

It is also worth noting that the low cost of access to W-MHIs was a perceived facilitator in a few of the included studies (i.e., 5 in YP, and 2 in healthcare providers). The majority of W-MHIs were delivered at a significantly low or even no cost compared to in-person services. This can partially explain why YP might overlook the impact of low cost when engaging with W-MHIs. The affordability of W-MHIs made it easier for YP to access mental health support, allowing users to gain some benefits from W-MHIs at a significantly lower cost [59]. However, the affordability of accessing services for users does not necessarily imply that the cost of maintaining quality services is also low. Resources are required for specialized training, clinical supervision, and ongoing support in implementing and maintaining online mental health care quality [129]. The provision of online mental health programs has been limited by a lagging infrastructure and skill base [130]. Thus, it is not surprising that healthcare providers in our review commonly reported insufficient organizational support (e.g., limited technical infrastructure, limited funding, and a lack of training for therapists) as an important barrier to providing W-MHIs. Our finding is consistent with Ivlev et al., that identified a lack of guidelines and tailored training to support therapists as the most frequent barrier to implementing digital CBT for adolescents with depression [26].

### Implication and future research

Our review identified numerous influencing factors to user engagement with W-MHIs. We found that users of different ages encountered different barriers/facilitators to the interventions. For future W-MHIs, it is crucial to actively engage YP at different ages as targeted users in the co-design and refinement process. Program development needs to focus on the informativeness and relevance of the content, ensure user safety, and improve the program attractiveness by using multimedia (e.g., text, pictures, videos). Furthermore, it is imperative to promote flexibility and connectedness in the W-MHIs and improve the personalization to better suit each individual user. Considering the pivotal role of health professionals and providers in implementing W-MHIs, there is a need to advocate for increased organizational support and awareness among professionals and providers regarding their respective roles. In addition, the mixed views regarding the absence of face-to-face contact in W-MHIs highlight the potential of blended mental health interventions (i.e., including both online and face-to-face sessions).

This review provides valuable insights into barriers/facilitators of user engagement. The lack of identified studies from LMICs suggests that more research is needed in the LMIC context. We found a lack of evaluation of the relative weight or importance of these factors on user engagement, as well as inconsistency in the definition of ‘engagement’ across the included studies. No studies targeted *‘effective engagement’*, whereby users might not engage sufficiently to accomplish the expected outcomes [14]. Instead, users may partially complete the program and potentially receive some benefits, yet not attaining the full benefits if they complete the entire program. Moreover, we found that cost did not appear to be a common driver for user engagement, although it was an important barrier to accessing in-person therapies [131, 132]. Therefore, further research is needed to (1) examine the impact of cost on user engagement with W-MHIs, (2) evaluate the impact of these barriers/facilitators on user engagement, (3) reach a consensus on ‘engagement’ definition and how effective engagement can be measured in online mental health care, and (4) identify factors influencing ‘effective engagement’ instead of ‘higher engagement’.

### Strengths and limitations

A major strength of this current review is the comprehensive inclusion of factors influencing users’ engagement with all web-based interventions for a wide range of mental problems. This inclusivity extends to perspectives from YP, their parents, and healthcare providers. We categorized and reported factors influencing engagement by the percentage of endorsement or the mean rating score in quantitative studies. This can improve the validation of qualitative findings. Nevertheless, there are some limitations. First, great heterogeneity across quantitative studies precluded meta-analysis. Second, we applied a subjective assessment of barriers/facilitators reported in quantitative findings, indicating that some factors were rated unfavorably but might not influence engagement. For example, websites were rated as cumbersome and required significant learning [76], that was indicated as a barrier in our review. However, YP might not perceive the same. Therefore, caution was taken in the data synthesis and interpretation by allocating quantitative findings into appropriate qualitative subthemes. Third, existing quantitative questionnaires focused more on facilitators than barriers. This might have given more weight to some facilitators, especially those related to interventions and practical factors. Lastly, this review only included English-published peer-reviewed journals from 2010. Therefore, any publications in other languages or prior to 2010 were not captured here. Gray literature was also excluded from this review to assure the quality of studies but there might be potential risk of bias.

## Conclusion

This review synthesized barriers and facilitators of engaging with W-MHIs from multiple perspectives, including YP, their parents, and healthcare providers. The results highlighted practical, intervention-related, and user-related factors that influence user engagement with W-MHIs. These factors should be considered in future W-MHI design to improve user engagement. Understanding these factors can narrow the gap between existing (and future) W-MHIs and unmet needs of users.

## Supplementary Information

Below is the link to the electronic supplementary material.

Supplementary file1 (PDF 97 KB)

Supplementary file2 (PDF 151 KB)

Supplementary file3 (PDF 129 KB)

Supplementary file4 (PDF 224 KB)

Supplementary file5 (PDF 217 KB)

## Data Availability

Not applicable.

## Code availability

The authors used Microsoft Word, Microsoft Excel, and EndNote.
