# Supplementary material for: Barriers to and facilitators of user engagement with web-based mental health interventions in young people: a systematic review
Source: Eur Child Adolesc Psychiatry. 2024 Feb 14;34(1):83–100. doi: 10.1007/s00787-024-02386-x (PMC11805866; doi:10.1007/s00787-024-02386-x)
Supplement: Supplementary file 1 — Supplementary file1 (PDF 97 KB) [file 787_2024_2386_MOESM1_ESM.pdf]

Journal: European Child and Adolescent Psychiatry

Article title: “Barriers to and facilitators of user engagement with web-based mental health intervention in young people: a systematic review”

Authors:
Thi Quynh Anh Ho; Long Khanh-Dao Le; Lidia Engel; Ngoc Le; Glenn Melvin; Ha N.D. Le*; Cathrine Mihalopoulos*

*: Joint senior authors

Corresponding author:
Thi Quynh Anh Ho – School of Health and Social Development, Deakin University, Melbourne, Victoria, Australia tqho@deakin.edu.au

Supplementary material 1 – Search terms on MEDLINE

Search strategy:

Adolescent & Young people block
AND Web-based intervention block
AND Mental health block
AND Barriers

Adolescents & Young people block

TI (adolescen* OR youth OR student* OR young OR teen*) OR AB (adolescen* OR youth OR student* OR young OR teen*) OR (MH "Adolescent") OR (MH "Young Adult")

Web-based interventions block

TI (digital N3 intervention*) OR TI (online N3 intervention*) OR TI (internet N3 intervention*) OR TI (web* N3 intervention*) OR TI (digital N3 treatment*) OR TI (online N3 treatment*) OR TI (internet N3 treatment*) OR TI (web* N3 treatment*) OR TI (digital N3 program*) OR OR TI (online N3 program*) OR OR TI (internet N3 program*) OR TI (web* N3 program*) OR TI (technolog* N3 intervention*) OR TI (web* N3 app*) OR TI (digital N3 therap*) OR TI (online N3 therap*) OR OR TI (internet N3 therap*) OR OR TI (web* N3 therap*) OR AB (digital N3 intervention*) OR AB (online N3 intervention*) OR AB (internet N3 intervention*) OR AB (web* N3 intervention*) OR AB (digital N3 treatment*) OR AB (online N3 treatment*) OR AB (internet N3 treatment*) OR AB (web* N3 treatment*) OR AB (digital N3 program*) OR AB (online N3 program*) OR AB (internet N3 program*) OR AB (web* N3 program*) OR AB (technolog* N3 intervention*) OR AB (web* N3 app*) OR AB (digital N3 therap*) OR AB (online N3 therap*) OR AB (internet N3 therap*) OR AB (web* N3 therap*) OR TI “digital health” OR TI “ehealth” OR TI “e-health” OR TI “digital mental health” OR TI “e-mental health” OR TI “eMental health” OR TI “online mental health” OR TI “electronic mental health” OR AB “digital health” OR AB “ehealth” OR AB “e-health” OR AB “digital mental health” OR AB “e-mental health” OR AB “eMental health” OR AB “online mental health” OR AB “electronic mental health” OR TI telemedicine OR AB telemedicine OR (MH "Internet-Based Intervention") OR (MH "Telemedicine")

Mental health block

TI “mental disorder*” OR TI “anxiety disorder*” OR TI “agoraphobia” OR TI “separation anxiety” OR TI “neurocirculatory asthenia” OR TI “neurotic disorder*” OR TI “obsessive-compulsive disorder” OR TI “hoarding disorder” OR TI “panic disorder” OR TI “phobic disorder*” OR TI “social phobia” OR TI “phobia*” OR TI “bipolar and related disorder*” OR TI “bipolar disorder” OR TI “bipolar” OR TI “disruptive disorder*” OR TI “impulse control disorder*” OR TI “conduct disorder*” OR TI “firesetting behavi?r” OR TI “gambling” OR TI “trichotillomania” OR TI “dissociative disorder*” OR TI “multiple personality disorder” OR TI “anorexia” OR TI “anorexia nervosa” OR TI “binge-eating disorder” OR TI “bulimia” OR TI “bulimia nervosa” OR TI “feeding and eating disorder*” OR TI “feeding disorder*” OR TI “eating disorder*” OR TI “female athlete triad syndrome” OR TI “pica” OR TI “depression” OR TI “mood disorder*” OR TI “postpartum

depression” OR TI “depressive disorder” OR TI “major depressive disorder” OR TI “treatment-resistant depressive disorder” OR TI “dysthymic disorder” OR TI “premenstrual dysphoric disorder” OR TI “seasonal affective disorder” OR TI “cyclothymic disorder” OR TI “neurocognitive disorder*” OR TI “alcohol amnestic disorder” OR TI “korsakoff syndrome” OR TI “auditory perceptual disorder*” OR TI “attention deficit behavi?r” OR TI “attention deficit and disruptive behavi?r disorder*” OR TI “attention deficit disorder with hyperactivity” OR TI “attention deficit hyperactivity disorder” OR TI “conduct disorder” OR TI “pervasive child development disorder*” OR TI “child development disorder*” OR TI “asperger syndrome” OR TI “autism spectrum disorder” OR TI “autistic disorder” OR TI “mutism” OR TI “reactive attachment disorder” OR TI “childhood schizophrenia” OR TI “schizophrenia” OR TI “catatonic schizophrenia” OR TI “disorganized schizophrenia” OR TI “paranoid schizophrenia” OR TI “stereotypic movement disorder” OR TI “tic disorder*” OR TI “tourette syndrome” OR TI “paraphilic disorder*” OR TI “exhibitionism” OR TI “fetishism” OR TI “masochism” OR TI “pedophilia” OR TI “sadism” OR TI “transvestism” OR TI “voyeurism” OR TI “personality disorder*” OR TI “antisocial personality disorder” OR TI “borderline personality disorder” OR TI “compulsive personality disorder” OR TI “dependent personality disorder” OR TI “histrionic personality disorder” OR TI “hysteria” OR TI “paranoid personality disorder” OR TI “passive-aggressive personality disorder” OR TI “hallucin*” OR TI “schizoid personality disorder” OR TI “schizotypal personality disorder” OR TI “affective disorder*” OR TI “capgras syndrome” OR TI “delusional parasitosis” OR TI “delusion*” OR TI “morgellons disease” OR TI “paranoid disorder*” OR TI “psychotic disorder*” OR TI “psychos?s” OR TI “substance-induced psychos?s” OR TI “alcohol-induced psychos?s” OR TI “shared paranoid disorder” OR TI “psychological sexual dysfunctions” OR TI “dyspareunia” OR TI “vaginismus” OR TI “sleep wake disorder*” OR TI “dyssomnia*” OR TI “sleep deprivation” OR TI “sleep disorder*” OR TI “circadian rhythm sleep disorders” OR TI “jet lag syndrome” OR TI “intrinsic sleep disorder*” OR TI “excessive somnolence disorder*” OR TI “sleep initiation and maintenance disorder*” OR TI “sleep initiation disorder*” OR TI “sleep maintenance disorder*” OR TI “parasomnia*” OR TI “nocturnal paroxysmal dystonia” OR TI “sleep arousal disorder*” OR TI “night terror*” OR TI “somnambulism” OR TI “sleep-wake transition disorder*” OR TI “somatoform disorder*” OR TI “body dysmorphic disorder*” OR TI “conversion disorder” OR TI “factitious disorder*” OR TI “munchausen syndrome” OR TI “hypochondriasis” OR TI “neurasthenia” OR TI “substance-related disorder*” OR TI “addicti*” OR TI “alcohol-related disorder*” OR TI “alcohol use disorder” OR TI “alcohol withdrawal delirium” OR TI “alcohol* intoxication” OR TI “alcoholism” OR TI “binge drinking” OR TI “wernicke encephalopathy” OR TI “amphetamine-related disorder*” OR TI “cocaine-related disorder*” OR TI “cocaine use” OR TI “inhalant abuse” OR TI “marijuana abuse” OR TI “opioid-related disorder*” OR TI “opioid misuse” OR TI “heroin dependence” OR TI “morphine dependence” OR TI “phencyclidine abuse” OR TI “substance abuse” OR TI “tobacco use disorder” OR TI “trauma and stressor related disorder*” OR TI “trauma related disorder*” OR TI “stressor related disorder*” OR TI “adjustment disorder*” OR TI “battered child syndrome” OR TI “combat disorder*” OR TI “psychological trauma” OR TI “post-traumatic stress disorder*” OR TI “ptsd” OR TI “acute stress disorder*” OR TI “traumatic stress disorder*” OR TI “acute traumatic stress disorder*” OR TI “isolat*” OR TI “lonel*” OR TI “mania” OR TI “manic” OR TI “neurosis” OR TI “neurotic” OR TI “overdos*” OR TI “parasuicid*” OR TI “para-suicid*” OR TI “suicid*” OR TI “selfcut*” OR TI “self-cut*” OR TI “self-harm” OR TI “selfharm*” OR TI “self-harm*” OR TI “selfinjur*” OR TI “self-injur*” OR TI “selfpoison*” OR TI “self-poison*” OR AB “mental disorder*” OR AB “anxiety disorder*” OR AB “agoraphobia” OR AB “separation anxiety” OR AB “neurocirculatory asthenia” OR AB “neurotic disorder*” OR AB “obsessive-compulsive disorder” OR AB “hoarding disorder” OR AB “panic disorder” OR AB “phobic disorder*” OR AB “social phobia” OR AB “phobia*” OR AB “bipolar and related disorder*” OR AB “bipolar disorder” OR AB “bipolar” OR AB “disruptive disorder*” OR AB “impulse control disorder*” OR AB “conduct disorder*” OR AB “firesetting behavi?r” OR AB “gambling” OR AB “trichotillomania” OR AB “dissociative disorder*” OR AB “multiple personality disorder” OR AB “anorexia” OR AB “anorexia nervosa” OR AB “binge-eating disorder” OR AB “bulimia” OR AB “bulimia nervosa” OR AB “feeding and eating disorder*” OR AB “feeding disorder*” OR AB “eating disorder*” OR AB “female athlete triad syndrome” OR AB “pica” OR AB “depression” OR AB “mood disorder*” OR AB “postpartum depression” OR AB “depressive disorder” OR AB “major depressive disorder” OR AB “treatment-resistant depressive disorder” OR AB “dysthymic disorder” OR AB “premenstrual dysphoric disorder” OR AB “seasonal affective disorder” OR AB “cyclothymic disorder” OR AB “neurocognitive disorder*” OR AB “alcohol amnestic disorder” OR AB “korsakoff syndrome” OR AB “auditory perceptual disorder*” OR AB “attention deficit behavi?r” OR AB “attention deficit and disruptive behavi?r disorder*” OR AB “attention deficit disorder with hyperactivity” OR AB “attention deficit hyperactivity disorder” OR AB “conduct disorder” OR AB “pervasive child development disorder*” OR AB “child development disorder*” OR AB “asperger syndrome” OR AB “autism spectrum disorder” OR AB “autistic disorder” OR AB “mutism” OR AB “reactive attachment disorder” OR AB “childhood schizophrenia” OR AB “schizophrenia” OR AB “catatonic schizophrenia” OR AB “disorganized schizophrenia” OR AB “paranoid schizophrenia” OR AB “stereotypic movement disorder” OR AB “tic disorder*” OR AB “tourette syndrome” OR AB “paraphilic disorder*” OR AB “exhibitionism” OR AB

“fetishism” OR AB “masochism” OR AB “pedophilia” OR AB “sadism” OR AB “transvestism” OR AB “voyeurism” OR AB “personality disorder*” OR AB “antisocial personality disorder” OR AB “borderline personality disorder” OR AB “compulsive personality disorder” OR AB “dependent personality disorder” OR AB “histrionic personality disorder” OR AB “hysteria” OR AB “paranoid personality disorder” OR AB “passive-aggressive personality disorder” OR AB “hallucin*” OR AB “schizoid personality disorder” OR AB “schizotypal personality disorder” OR AB “affective disorder*” OR AB “capgras syndrome” OR AB “delusional parasitosis” OR AB “delusion*” OR AB “morgellons disease” OR AB “paranoid disorder*” OR AB “psychotic disorder*” OR AB “psychos?s” OR AB “substance-induced psychos?s” OR AB “alcohol-induced psychos?s” OR AB “shared paranoid disorder” OR AB “psychological sexual dysfunctions” OR AB “dyspareunia” OR AB “vaginismus” OR AB “sleep wake disorder*” OR AB “dyssomnia*” OR AB “sleep deprivation” OR AB “sleep disorder*” OR AB “circadian rhythm sleep disorders” OR AB “jet lag syndrome” OR AB “intrinsic sleep disorder*” OR AB “excessive somnolence disorder*” OR AB “sleep initiation and maintenance disorder*” OR AB “sleep initiation disorder*” OR AB “sleep maintenance disorder*” OR AB “parasomnia*” OR AB “nocturnal paroxysmal dystonia” OR AB “sleep arousal disorder*” OR AB “night terror*” OR AB “somnambulism” OR AB “sleep-wake transition disorder*” OR AB “somatoform disorder*” OR AB “body dysmorphic disorder*” OR AB “conversion disorder” OR AB “factitious disorder*” OR AB “munchausen syndrome” OR AB “hypochondriasis” OR AB “neurasthenia” OR AB “substance-related disorder*” OR AB “addicti*” OR AB “alcohol-related disorder*” OR AB “alcohol use disorder” OR AB “alcohol withdrawal delirium” OR AB “alcohol* intoxication” OR AB “alcoholism” OR AB “binge drinking” OR AB “wernicke encephalopathy” OR AB “amphetamine-related disorder*” OR AB “cocaine-related disorder*” OR AB “cocaine use” OR AB “inhalant abuse” OR AB “marijuana abuse” OR AB “opioid-related disorder*” OR AB “opioid misuse” OR AB “heroin dependence” OR AB “morphine dependence” OR AB “phencyclidine abuse” OR AB “substance abuse” OR AB “tobacco use disorder” OR AB “trauma and stressor related disorder*” OR AB “trauma related disorder*” OR AB “stressor related disorder*” OR AB “adjustment disorder*” OR AB “battered child syndrome” OR AB “combat disorder*” OR AB “psychological trauma” OR AB “post-traumatic stress disorder*” OR AB “ptsd” OR AB “acute stress disorder*” OR AB “traumatic stress disorder*” OR AB “acute traumatic stress disorder*” OR AB “isolat*” OR AB “lonel*” OR AB “mania” OR AB “manic” OR AB “neurosis” OR AB “neurotic” OR AB “overdos*” OR AB “parasuicid*” OR AB “para-suicid*” OR AB “suicid*” OR AB “selfcut*” OR AB “self-cut*” OR AB “self-harm” OR AB “selfharm*” OR AB “self-harm*” OR AB “selfinjur*” OR AB “self-injur*” OR AB “selfpoison*” OR AB “self-poison*” OR TI "substance use" OR AB "substance use" OR (TI "psychological well-being" OR AB "psychological well-being") OR (TI "psychological wellbeing" OR AB "psychological wellbeing") OR (TI "mental well-being" OR AB "mental well-being") OR (TI "mental wellbeing" OR AB "mental wellbeing") OR TI "mental* ill*" OR AB "mental* ill*" OR TI "mental health" OR AB "mental health" OR TI "mindfulness" OR AB "mindfulness" OR (MH "Mental Health") OR (MH "Mental Disorders+")

**Barrier block**

TI (acceptance OR barrier* OR engagement OR acceptability OR attitude* OR adherence OR "qualitative") OR AB (acceptance OR barrier* OR engagement OR acceptability OR attitude* OR adherence OR "qualitative") OR (MH "Qualitative Research") OR (MH "Patient Satisfaction+") OR (MH "Patient Acceptance of Health Care+")
