# Supplementary material for: Barriers to and facilitators of user engagement with web-based mental health interventions in young people: a systematic review
Source: Eur Child Adolesc Psychiatry. 2024 Feb 14;34(1):83–100. doi: 10.1007/s00787-024-02386-x (PMC11805866; doi:10.1007/s00787-024-02386-x)
Supplement: Supplementary file 2 — Supplementary file2 (PDF 151 KB) [file 787_2024_2386_MOESM2_ESM.pdf]

Journal: European Child and Adolescent Psychiatry

Article title: "Barriers to and facilitators of user engagement with web-based mental health intervention in young people: a systematic review"

Authors:
Thi Quynh Anh Ho; Long Khanh-Dao Le; Lidia Engel; Ngoc Le; Glenn Melvin; Ha N.D. Le*; Cathrine Mihalopoulos*

*: Joint senior authors

Corresponding author:
Thi Quynh Anh Ho – School of Health and Social Development, Deakin University, Melbourne, Victoria, Australia
tqho@deakin.edu.au

Supplementary material 2 – Study characteristics

First author, year	Participants	Health condition of users (baseline)	Study design	Method used to collect user feedback	Country of study
**Anttila 2019**	167 adolescents (15-19 years, 68.3% female);<br>10 teachers (program tutors for the adolescents)	-	Quantitative	Questionnaires (rating)<br>Yes/no questions<br>Open-ended questions	Thailand
**Anttila 2020**	46 adolescents (15-17 years, 74% female)	Depression or anxiety symptoms (with or without relevant diagnosis)	Mixed method	Questionnaires (rating)<br>Open-ended questions (Interview/post-online survey)	Finland
**Aspvall 2020**	31 adolescents (8-18 years, 67.7% female);<br>6 therapists	Diagnosis of OCD (based on DSM-5 or ICD-10)	Quantitative	Open-ended feedback questions	Sweden<br>UK<br>Australia
**Babiano-Espinosa 2021**	25 children (8-17 years, 56% female);<br>25 parents	Diagnosis of OCD (based on DSM-5)	Quantitative	Questionnaires (rating)	Norway
**Bailey 2021**	15 young people (17-24 years, 60% female)	Having suicidal ideation	Qualitative	Semi-structured interview	Australia
**Bannink 2014**	1,087 adolescents (15.9 years, 42.87% female);<br>nurses	-	Quantitative	Questionnaires (rating)	Netherlands
**Banwell 2022**	Children and young people (10-25 years, 75.3% female)	N/A	Quantitative	Questionnaires (rating)	UK
**Bautista 2022**	35 college students (19-24 years, 71.4% female);<br>5 coaches	Self-reported social anxiety (Mini-SPIN)	Quantitative	Questionnaires (rating)	US
**Beames 2021**	201 secondary school staffs and counselors	-	Quantitative	Survey	Australia
**Benjet 2020**	7,849 first year university students	-	Quantitative	Questionnaires (rating)	Mexico

First author, year	Participants	Health condition of users (baseline)	Study design	Method used to collect user feedback	Country of study
	(19.37 years, 54.73% female)				
**BevanJones 2020**	43 adolescents (13-23 years, 79% female); 31 parents/carers; 13 professionals	Current or past depression symptoms; or at elevated risk of depression	Mixed methods	Interview with young people/parents/cares Focus group with professionals Questionnaires (rating)	UK
**Bohleber 2016**	670 adolescents (16.5-18.4 years, 47.4% female)	-	Mixed methods	Survey (feedback) Semi structured interview	Switzerland
**Bowman 2020**	9 LGBT young adults (18-25 years); 6 service providers	-	Qualitative	Semi structured interview	Australia
**Bradley 2012**	13 adolescents (15-18 years, 69.23% female)	No previous mental illness diagnosis	Qualitative	Semi-structured interview	Canada
**Bunnell 2017**	1,997 adolescents (12-17 years, 49.4% female); their families	Disaster-affected	Quantitative	Interview	US
**Calear 2013**	1,477 adolescents (12-17 years, 55.92% female)	-	Quantitative RCT	Questionnaires	Australia
**Chan 2016**	19 university students (19-24 years, 52.63% female)	-	Qualitative	Focus group	Australia
**Clark 2018**	29 male adolescents (12-18 years)	-	Qualitative	Interview (individual and focus group)	Australia
**Dai 2022**	120 nursing students (Mean age 19.16 years, 79.63% female)	N/A	Quantitative	Questionnaires (rating)	China
**Dobias 2022**	1,194 teenagers (13-19 years; 35.09% female and 24.01% other)	N/A	Quantitative	Questionnaires (rating) Writing feedback	US
**Ellis 2013**	704 young men (16-24 years)	-	Mixed method	Questionnaires (rating) Focus group	Australia
**Geirhos 2022**	30 young people (12-21 years; 73% female)	Cystic fibrosis, juvenile idiopathic arthritis, or type-1 diabetes (self-reported)	Quantitative RCT	Feedback	Germany
**Gericke 2021**	9 university students (17-20 years, 66.67% female)	Self-reported moderate to moderately severe symptom of depression (based on PHQ-9)	Qualitative	Semi-structured interview	South Africa
**González-García 2021**	66 first year students (18-25 years, 86.36% female)	-	Quantitative	Survey	Spain

First author, year	Participants	Health condition of users (baseline)	Study design	Method used to collect user feedback	Country of study
**Hämäläinen 2021**	157 ninth-grade adolescents (15 years, 50% female)	-	Quantitative	Questionnaires (rating)	Finland
**Iloabachie 2011**	83 adolescents (14-21 years, 56% female); parents of those below 18 years	Reporting one core symptom of depression	Mixed method	Questionnaire (rating) Interview/Comments from the intervention	US
**Kahl 2020**	1,609 young people (16-25 years, 84% female)	-	Quantitative (longitudinal)	Questionnaires (rating)	Australia
**Kanuri 2020**	15 students (18-22 years, 13.3% female)	Generalized anxiety disorder	Mixed methods	Questionnaires (rating) Semi-structured focus group	India
**Karim 2021**	66 adolescents and young adults (14-26 years)	Self-reported current or prior symptoms of depression or anxiety	Mixed methods	Questionnaires (rating) Open-ended questions	US
**Karyotaki 2022**	100 college students (21.91 years ± 2.61, 81% female)	Mild to moderate depression and/ or anxiety symptoms (self-reported PHQ-9 & GAD-7)	Quantitative RCT	Questionnaires	Netherlands
**Kurki 2018**	9 nurses	-	Qualitative	Interview	Finland
**Lattie 2017**	39 high school students (14-19 years, 74.4% female)	Self-reported symptom of depression (based on CES-D) or use of cigarettes or alcohol (based on YRBS)	Mixed methods	Questionnaire Interview	US
**Leech 2020**	161 young adults (18-24 years, 71.4% female)	-	Quantitative	Questionnaires (rating)	Australia
**Lenhard 2016**	8 adolescents (12-17 years, 50% female)	Diagnosis of OCD	Qualitative	Interview	Sweden
**Lilja 2021**	14 adolescents (13-18 years, 93% female); 14 parents	Mild to moderate anxiety problems	Mixed method	Interview	Sweden
**Lillevoll 2014**	775 high school students (15-20 years, 56.8% female)	-	Quantitative RCT	Questionnaires (rating)	Norway
**Lindegaard 2022**	15 refugee youth (15-26 years, 7% female)	Elevated depression and/or anxiety symptoms (self-reported HSCL-25)	Mixed method	Interview	Sweden
**Mamdouh 2022**	707 students (18-25 years, 60.5% female)	N/A	Quantitative	Questionnaires (yes/no, rating, qualitative)	Egypt

First author, year	Participants	Health condition of users (baseline)	Study design	Method used to collect user feedback	Country of study
**Manicavasagar 2014**	154 young people (12-18 years, 67.5% female)	-	Mixed method (RCT & qualitative)	Questionnaires (rating) Open-ended question	Australia
**Marko 2010**	83 adolescents (14-21 years, 57% female)	Self-reported one core symptom of depression	Quantitative	Survey (rating) & observation	US
**Mawdsley 2022**	43 young people (18-25 years, mostly female); 8 counsellors	N/A	Qualitative	Interview	UK
**McDanal 2022**	258 LGBT+ youths (11-17 years)	N/A	Mixed method	Questionnaires (rating) Open-ended questions	US
**Nicolaou 2022**	62 young women (13-25 years)	Showing early signs of an eating disorder or at-risk for developing an eating disorder (self-reported WCS)	Quantitative	Questionnaires (rating) Open-ended questions	Cyprus
**Mar 2014**	23 young people (18-24 years, 95.65% female)	Self-reported current or lifetime depression or anxiety, and having lifetime suicidal ideations	Qualitative	Interview	Canada
**O'Bree 2021**	70 young people (14-25 years, 47.14% female)	Social anxiety	Qualitative	Semi-structured interview	Australia
**Pine 2020**	98 teachers and health professionals	-	Qualitative	Open-ended questions	New Zealand
**Păsărelu 2021**	15 adolescents (12-16 years, 66.6% female)	Diagnosed with anxiety and or depressive disorders	Quantitative	Questionnaires (rating) Open-ended questions	Romania
**Pretorius 2010**	11 young people (16-20 years, 100% female)	Bulimia nervosa or atypical bulimia nervosa (based on DSM-IV)	Qualitative	Semi-structured interview	UK
**Price 2015**	2,000 adolescents (12-17 years, 51% female)	Exposed to a series of tornados	Quantitative	Observation	US
**Punukollu 2020**	31 pupils (11-14 years); 30 teachers	-	Qualitative	Focus group	Scotland
**Richiello 2022**	8 counsellors	N/A	Qualitative	Interview	UK
**Rickwood 2019**	2,280 young people (12-25 years, 77.76% female)	-	Quantitative	Questionnaires (rating)	Australia

First author, year	Participants	Health condition of users (baseline)	Study design	Method used to collect user feedback	Country of study
**Sansom-Daly 2019**	45 young people<br>(15-25 years, 51.1% female);<br>19 caregivers	At the end of curative cancer treatment completion	Mixed method	Questionnaires (rating)<br>Open-ended question	Australia
**Santesteban-Echarri 2017**	38 young people<br>(15-26 years, 50% female)	Diagnosis of MDD (based on DSM-IV) and remission for MDD	Qualitative	Semi-structured interview<br>Follow up focus group	Australia
**Sawrikar 2022**	248 young people<br>(17-25 years, 41% female, 0.4% other)	N/A	Quantitative	Questionnaires (rating)	UK
**Schleider 2020**	694 youths<br>(11-17 years (including six under 10 years), 78.1% female)	-	Quantitative	Questionnaires (rating)	Australia
**Schmitt 2022**	24 adolescents<br>(12-17years, 50% female);<br>24 parents	Showing increased anxiety and/or depression symptoms (self-reported RCADS-30)	Quantitative	Questionnaires (rating)	Spain
**Shandley 2010**	266 people<br>(18-25 years, 66.17% female)	-	Quantitative	Online survey (rating, yes/no)	Australia
**Smart 2021**	13 adolescents<br>(13-17 years, 76.92% female)	Primary diagnosis of anxiety disorder (based on DMS-IV)	Qualitative	Semi-structured interview	UK
**Sobowale 2016**	20 youths (15-21 years, 50% female);<br>20 parents	-	Qualitative	Focus group	Vietnam
**Sweeney 2016**	217 adolescents<br>(13–18 years, 71.9% female)	-	Quantitative	Questionnaires (rating)	Australia
**vanDalen 2022**	14 adolescents<br>(12-17 years, 28.6% female)	Self-reported visible difference due to any injury or condition	Mixed method	Interview	Netherlands
**Watkins 2017**	8 black men<br>(18-24 years)	Never been diagnosed a mental condition	Qualitative	In-depth interview	US
**Weineland 2020**	14 primary care therapists	-	Qualitative	Semi-structured interview	Sweden
**Wetterlin 2014**	521 youth<br>(17-24 years, 76.6% female)	-	Quantitative	Questionnaires (rating)	Canada
**Windler 2019**	96 adolescents and young adults<br>(14-26 years, 75% female);<br>14 moderators	Depression or anxiety (self-reported)	Qualitative	Blog posts + comments (by moderators and users)<br>Interview (with moderators)	US

First author, year	Participants	Health condition of users (baseline)	Study design	Method used to collect user feedback	Country of study
**Woolderink 2015**	13 adolescents (16-25 years, 92% female); 4 providers	Their parents had mental health problems or addictions	Qualitative	Semi-structured interview	Netherlands
**Wuthrich 2021**	15 adolescents (12-18 years, 73.3% female; 10 clinicians	Anxiety (based on the intake assessment)	Mixed method	Questionnaires (rating) and free response	Australia
**Zeiler 2021**	29 pupils (14-19 years, 62.1% female); 59 stakeholders (i.e., teachers, school psychologists and policy makers)	-	Mixed method	Semi-structured interview<br>Focus group<br>Questionnaires (rating)	Austria<br>Spain

Note: MDD = Major depressive disorder; CES-D = The Center for Epidemiological Studies-Depression [1]; DSM-IV = The Diagnostic and Statistical Manual of Mental Disorder, Fourth Edition [2]; DSM-5 = The Diagnostic and Statistical Manual of Mental Disorder, Fifth Edition [3]; GAD-7 = Generalized Anxiety Disorder 7-Item Scale [4]; HSCL-25 = Hopkins Symptom Checklist-25 [5]; ICD-10 = The International Classification of Diseases, Tenth Revision [6]; Mini-SPIN = Mini-Social Phobia Inventory [7]; N/A = Not applicable; OCD = Obsessive-compulsive disorder; PHQ-9 = Patient Health Questionnaire – 9 Item [8]; RCADS-30 = Revised Child's Anxiety and Depression Scale [9]; WCS = Weight Concern Scale [10]; YRBS = Youth Risk Behavior Survey [11].

References:

1. Radloff LS (1977) The CES-D Scale:A Self-Report Depression Scale for Research in the General Population. Applied Psychological Measurement 1:385-401.https://doi.org/10.1177/014662167700100306
2. American Psychiatric Association (1994) Diagnostic and statistical manual of mental disorders: DSM-IV. American psychiatric association Washington, DC
3. American Psychiatric Association (2013) Diagnostic and statistical manual of mental disorders: DSM-5. American psychiatric association Washington, DC
4. Spitzer RL, Kroenke K, Williams JB, Löwe B (2006) A brief measure for assessing generalized anxiety disorder: the GAD-7. Arch Intern Med 166:1092-1097.https://doi.org/10.1001/archinte.166.10.1092
5. Nettelbladt P, Hansson L, Stefansson CG, Borgquist L, Nordström G (1993) Test characteristics of the Hopkins Symptom Check List-25 (HSCL-25) in Sweden, using the Present State Examination (PSE-9) as a caseness criterion. Social Psychiatry and Psychiatric Epidemiology 28:130-133.https://doi.org/10.1007/BF00801743
6. World Health Organization (1992) The ICD-10 classification of mental and behavioural disorders: clinical descriptions and diagnostic guidelines. World Health Organization
7. Connor KM, Kobak KA, Churchill LE, Katzelnick D, Davidson JR (2001) Mini-SPIN: A brief screening assessment for generalized social anxiety disorder. Depress Anxiety 14:137-140.https://doi.org/10.1002/da.1055
8. Spitzer RL, Williams JB, Kroenke K, Hornyak R, McMurray J (2000) Validity and utility of the PRIME-MD patient health questionnaire in assessment of 3000 obstetric-gynecologic patients: the PRIME-MD Patient Health Questionnaire Obstetrics-Gynecology Study. Am J Obstet Gynecol 183:759-769.https://doi.org/10.1067/mob.2000.106580
9. Sandín B, Chorot P, Valiente RM, Chorpita BF (2010) Desarrollo de una versión de 30 items de la Revised Child Anxiety and Depression Scale. Revista de Psicopatología y Psicología Clínica 15:165-178.https://doi.org/10.5944/rppc.vol.15.num.3.2010.4095
10. Killen JD, Taylor CB, Hayward C, Haydel KF, Wilson DM, Hammer L, Kraemer H, Blair-Greiner A, Strachowski D (1996) Weight concerns influence the development of eating disorders: a 4-year prospective study. J Consult Clin Psychol 64:936-940.https://doi.org/10.1037//0022-006x.64.5.936
11. Centers for Disease Control and prevention (CDC) (2015) Youth Risk Behavior Survey.
