# Supplementary material for: Barriers to and facilitators of user engagement with web-based mental health interventions in young people: a systematic review
Source: Eur Child Adolesc Psychiatry. 2024 Feb 14;34(1):83–100. doi: 10.1007/s00787-024-02386-x (PMC11805866; doi:10.1007/s00787-024-02386-x)
Supplement: Supplementary file 3 — Supplementary file3 (PDF 129 KB) [file 787_2024_2386_MOESM3_ESM.pdf]

Journal: European Child and Adolescent Psychiatry

Article title: "Barriers to and facilitators of user engagement with web-based mental health intervention in young people: a systematic review"

Authors:
Thi Quynh Anh Ho; Long Khanh-Dao Le; Lidia Engel; Ngoc Le; Glenn Melvin; Ha N.D. Le*; Cathrine Mihalopoulos*

*: Joint senior authors

Corresponding author:
Thi Quynh Anh Ho – School of Health and Social Development, Deakin University, Melbourne, Victoria, Australia
tqho@deakin.edu.au

Supplementary material 3 – Intervention characteristics

Author, year	Name of Intervention	Therapeutic approach	Target mental health (primary outcome)	Human support	Length of the intervention
Anttila 2019	DepisNet-Thai	CBT	Depression, stress	Teacher (acting as a tutor) guided	5 modules (50mins each)
Anttila 2020	Depis.Net	CBT	Depression	Research nurse (expert)	5 sessions (45 mins each)
Aspvall 2020	BIP OCD	CBT	OCD	Therapist	12 modules for adolescents and 5 parallel modules for parents
Babiano-Espinosa 2021	Enhanced CBT (eCBT) package	CBT	OCD	Therapist	10 face-to-face and 12 webcam sessions
Bailey 2021	Affinity	Positive psychology and social networking	Suicidal ideation, depression, mindfulness, self-compassion, problem solving	Clinical experts or young people with lived experience of mental ill health	8 weeks
Bannink 2014	E-health4Uth Intervention	Psychoeducation and counseling	Health behavior and wellbeing (e.g., mental health, suicidal thoughts, suicide attempts)	Nurse	4 months
Banwell 2022	Kooth community	Social networking	Mental health and wellbeing	Peers	Not reported
Bautista 2022	Social phobia - This Way Up	CBT	Social anxiety	Peer coach	6 modules in 6-10 weeks
Beames 2021	Hypothetical intervention	CBT	Depression	Supervised by school staffs	7 sessions (20 mins each)
Benjet 2020	-	-	General mental health wellbeing	-	-

Author, year	Name of Intervention	Therapeutic approach	Target mental health (primary outcome)	Human support	Length of the intervention
BevanJones 2020	MoodHwb	CBT, positive psychology, interpersonal therapy, and family systems therapy	Depression, anxiety	Self-help	2 months
Bohleber 2016	Companion App	Psychoeducation and social networking	Stress	Psychologist or social worker	10 months
Bowman 2020	-	-	General mental health wellbeing	-	-
Bradley 2012	Feeling Better program	CBT	Depression, anxiety, and stress.	Self-help	12 modules
Bunnell 2017	Bounce Back Now	CBT	PTSD, depression	Self-help	4 modules
Calear 2013	MoodGYM	CBT	Anxiety and depression	Self-help	5 modules
Chan 2016	Virtual mental health clinic	-	General mental health wellbeing	-	-
Clark 2018	Chilled Out	CBT	Anxiety	Therapist	8 modules (30mins each)
Dai 2022	Mindfulness Living With Challenge (MLWC)	CBT	Depression, anxiety, stress	Research team (delivering, supervising during the course)	6 weeks
Dobias 2022	Koko minicourses	CBT	Hopelessness, self-hate, and desire to discontinue self-harm	Self-help	3 single sessions (5-8mins each)
Ellis 2013	-	-	General mental health wellbeing	-	-
Geirhos 2022	youthCOACH CD	CBT	Anxiety and depression	Self-guided or eCoach (master students, supervised by psychotherapists)	7 modules (50-70mins each)
Gericke 2021	ICare	CBT	Major depressive disorder and generalized anxiety disorder.	eCoach	7 sessions + 1 booster session
González-García 2021	E-Mindfulness intervention	CBT	Stress, anxiety, and self-compassion	Psychologists (delivering and supervising the intervention)	4 modules

**Author, year**	**Name of Intervention**	**Therapeutic approach**	**Target mental health (primary outcome)**	**Human support**	**Length of the intervention**
Hämäläinen 2021	Youth Compass	CBT	Stress and depression	Coach (bachelor or master psychology student)	5 modules
Iloabachie 2011	CATCH-IT	CBT and interpersonal therapy	Depression	Physicians (encouraging users to actively participate)	6 weeks
Kahl 2020	Reach Out	Psychoeducation	Depression, anxiety, Stress, and suicide risk	Self-help	12 weeks
Kanuri 2020	Mana Maali Digital Anxiety Program	CBT	Generalized anxiety disorder	Counsellors	20 sessions (10-15mins each)
Karim 2021	SOVA Blogging Ambassador intervention	Psychoeducation and social networking	Depression, anxiety	Advisor and moderator	3 months
Karyotaki 2022	ICare Prevent	CBT	Anxiety and depression	eCoach (master students, supervised by a senior researcher)	7 sessions
Kurki 2018	Depis.Net	CBT	Depression	Nurse	5 modules
Lattie 2017	ProjectTECH	CBT	Depression, stress	Peer or clinicians	40 lessons (5mins each)
Leech 2020	-	-	General mental health wellbeing	-	-
Lenhard 2016	BiP OCD	CBT	OCD	Clinician<br>Parents	12 chapters designed for the adolescent and 5 chapters designed for the parents.
Lilja 2021	"Anxiety Help for Adolescents"	CBT	Anxiety and depression	Therapist	8 modules
Lillevoll 2014	MoodGYM	CBT	psychological distress	Self-help	5 modules (45-60 mins each)
Lindegaard 2022	ICBT	CBT	Anxiety and depression	Therapist	10 modules
Mamdouh 2022	-	-	General mental health wellbeing	-	-
Manicavasagar 2014	Bite Back	Positive psychology	Depression, anxiety, and stress.	Self-help	6 weeks
Mar 2014	-	-	General mental health wellbeing	-	-
Marko 2010	CATCH-IT	CBT	Depression	Physicians (encouraging users to actively participate)	6 weeks

Author, year	Name of Intervention	Therapeutic approach	Target mental health (primary outcome)	Human support	Length of the intervention
Mawdsley 2022	not reported	Varied (e.g., CBT, person-centered approach)	General mental health wellbeing	Counsellors or psychotherapist	8 sessions (50 mins each)
McDanal 2022	Project YES (Youth Empowerment & Support)	CBT	Hopelessness, self-hate, perceived agency	Self-help	3 single sessions (30mins each)
Nicolaou 2022	AcceptME	CBT	Eating disorder	Self-help	6 sessions (30mins each)
O'Bree 2021	Entourage	CBT and social networking	Social anxiety	Clinicians	12 modules
Păsărelu 2021	REBT	CBT	Anxiety and depression	Psychotherapists	9 modules (over 6 weeks)
Pine 2020	-	-	General mental health wellbeing	-	-
Pretorius 2010	Web-based CBT intervention	CBT	Bulimia nervosa	Therapist	8 sessions
Price 2015	Bounce Back Now	CBT	Post-disaster mental health symptoms	Self-help	4 modules
Punukollu 2020	SafeSpot	Psychoeducation	Emotional distress	Safespotter (trained older pupil) and teachers	Not reported
Richiello 2022	Webchat on Amazon Web Services	Not reported	General mental health wellbeing	Counsellors	4-8 sessions (60mins each)
Rickwood 2019	eheadspace	Not reported	General mental health wellbeing	Clinicians	Not reported
Sansom-Daly 2019	Recapture Life	CBT	Stress	Facilitators (psychologists) -led small group discussion<br><br>A support person (parent, spouse) to participate (optional)	6 sessions
Santesteban-Echarri 2017	Rebound	Positive psychology	Depression	Moderators (clinicians) and expert peers	12 weeks
Sawrikar 2022	-	-	General mental health wellbeing	-	-

Author, year	Name of Intervention	Therapeutic approach	Target mental health (primary outcome)	Human support	Length of the intervention
Schleider 2020	Project YES (Youth Empowerment & Support)	CBT	Internalizing distress	Self-help	3 sessions (30mins each)
Schmitt 2022	Aprende a Manejar tus Emociones; Learn to Manage your Emotions (AMTE)	CBT	Anxiety and depression	Therapist	8 modules (30mins each)
Shandley 2010	Reach Out Central (ROC)	CBT	Depression, anxiety	Coach (narrator) (acting as a guide/mentor)	4 weeks
Smart 2021	BRAVE for Teenagers ONLINE	CBT	Anxiety disorder	Therapist	10 weekly sessions (60mins each) plus 2 booster sessions
Sobowale 2016	-	-	General mental health wellbeing	-	-
Sweeney 2016	-	-	General mental health wellbeing	-	-
vanDalen 2022	Face IT voor jongeren	CBT	Anxiety and depression (appearance-related distress)	Self-help (psychologists monitoring the progress)	8 lessons
Watkins 2017	YBMen Facebook	Psychoeducation and social networking	Subclinical distress and depression	Study team (posting prompts to group)	5 weeks
Weineland 2020	Anxiety Help for Adolescents	CBT	Anxiety	Therapist	8-10 weeks
Wetterlin 2014	-	Psychoeducation	General mental health wellbeing	-	-
Windler 2019	Supporting Our Valued Adolescents (SOVA)	Social networking	Depression, anxiety	Moderators	Not reported
Woolderink 2015	Kopstoring course	Psychoeducation	General mental health wellbeing	Psychologists/social workers (supervising the program)	8 weeks

**Author, year**	**Name of Intervention**	**Therapeutic approach**	**Target mental health (primary outcome)**	**Human support**	**Length of the intervention**
Wuthrich 2021	Chilled Out & Cool Kids program	CBT	Anxiety	Clinicians	8 sessions of internet-based CBT and 10 sessions of face-to-face CBT
Zeiler 2021	ICare intervention		General mental health wellbeing	-	-

Note: CBT = Cognitive Behavioral Therapy; PTSD = Post-traumatic stress disorder; OCD = Obessive-compulsive disorder
