# Supplementary material for: Barriers to and facilitators of user engagement with web-based mental health interventions in young people: a systematic review
Source: Eur Child Adolesc Psychiatry. 2024 Feb 14;34(1):83–100. doi: 10.1007/s00787-024-02386-x (PMC11805866; doi:10.1007/s00787-024-02386-x)
Supplement: Supplementary file 4 — Supplementary file4 (PDF 224 KB) [file 787_2024_2386_MOESM4_ESM.pdf]

Journal: European Child and Adolescent Psychiatry

Article title: "Barriers to and facilitators of user engagement with web-based mental health intervention in young people: a systematic review"

Authors:
Thi Quynh Anh Ho; Long Khanh-Dao Le; Lidia Engel; Ngoc Le; Glenn Melvin; Ha N.D. Le*; Cathrine Mihalopoulos*

*: Joint senior authors

Corresponding author:
Thi Quynh Anh Ho – School of Health and Social Development, Deakin University, Melbourne, Victoria, Australia
tqho@deakin.edu.au

Supplementary material 4 – Overall findings about barriers and facilitators of engagement with W-MHIs

Author, year	Participants	Barriers	Facilitators
Anttila 2019	Young people	Perceived unhelpfulness (harmful: 4%)	Ease of use (67%)<br>Perceived usefulness (27%)
	Teachers	Perceived unhelpfulness for users (harmful: 20%)	Easy to use (50%)<br>Perceived usefulness for users (40%)
Anttila 2020	Young people	Lack of time (busyness)<br>Technical problems (connection issues)<br>Perceived unhelpfulness (hard to portray feelings in writing; basic information, overall unhelpful)<br>Lack of fit (age-inappropriateness, moderator's approach, difficulty of tasks)<br>Limited support (long delay of support)<br>Unfavorable program design (textual, unclear structure of instructions; the way using pictures, 15.2%)<br>Lack of motivation (poor health condition, lack of mental health knowledge, disinterest)<br>Concern about privacy and confidentiality (29%)	Convenience (working at own pace, at anywhere, 95%)<br>Ease of use* (78%)<br>Perceived usefulness (effective content, self-expression and understand themselves)<br>Perceived fit (autonomy)<br>Connectedness (instant support)<br>Program design (attractive design, , clear, favorable features (reminders); easy to navigate, sufficient instructions, 15.2-84%)<br>Positive attitude (find W-MHIs acceptable)<br>Belief about its credibility<br>Feeling of safety (89%)
Aspvall 2020	Therapists	Practical factor (hard to get a detailed assessment of how much some patients adhered to treatment)<br>Intervention-related factor (written text - difficult to support users)<br>Concern about its effectiveness (less suitable for patients with more complex symptoms)	Convenience ('time-efficient')<br>Logistic factor (therapist training)<br>Intervention-related factors (guidelines regarding which patients be best suited to ICBT, routine to monitor risk, parents' involvement)
Babiano-Espinosa 2021	Young people	Lack of fit (age-inappropriateness)<br>Perceived unhelpfulness (25%)<br>Unfavorable program design (low attractiveness)	Perceived usefulness (58-83%)

Author, year	Participants	Barriers	Facilitators
	Parents (as part of the program)	Unhelpful features (evaluation questions, reminders: 13%)	Perceived usefulness (61-82%)
Bailey 2021	Young people	Lack of fit (moderator's approach)<br>Limited support (inability to interact closely with other users on a longer-term basic)<br>Unfavorable features (prohibiting of sharing information)<br>Concern about the safety (causing harm, unnecessarily worry)<br>Social factors (few active users)	Perceived fit (individualization)<br>Connectedness (connect with others, instant support)<br>Feeling of safety (non-judgment)
Bannink 2014	Young people	Perceived unhelpfulness (not containing new information, not giving insights into own behavior)<br>Unfavorable program design (low attractiveness)<br>Lack of motivation (lack of interest)	Ease of use (66.1%)<br>Perceived usefulness (leaning a lot, giving insights into own behaviors)<br>Program design (the way to get information, language is easy to understand, 83.4%)<br>Perceived fit (relevance, 42.2%)<br>Connectedness (support)<br>Sociodemographic (Non-Dutch: higher adherence, pre-university education: finding it easier to understand/easier to use/view websites more often, female: higher satisfaction)<br>Trust (information credibility)
	Nurses		Intervention-related factor (helpful information about the adolescents prior to the consultation)
Banwell 2022	Young people	Perceived unhelpfulness (12%)	Perceived usefulness (74.6%)<br>Sociodemographic (age, gender)
Bautista 2022	Young people		Trust (perceived credibility)<br>Connectedness (rapport with coaches)
	Coaches	Intervention-related factor (tasks were not applicable)	
Beames 2021	School staffs and counselors	Lack of time<br>Logistical factors (incompatibility between program and school values, limited resources, hard to get organizational support)<br>Provider-related factors (lack of mental health knowledge, rejection of their role in delivering program)<br>Intervention-related factors (concern about the online format, and privacy for users)<br>Perceived unhelpfulness for young people	Practical factors (easy to integrate into school curriculum)<br>Logistical factors (organizational support, available training)<br>Young people-related factors (mental health condition, perceived need, low cost to access)<br>Intervention-related factors (desirable features)

Author, year	Participants	Barriers	Facilitators
Benjet 2020	Young people	Low perceived need (unsure where to get help)<br>Uncertainty about treatment efficacy<br>Stigma (not want parents to know about their problems)	Features (reminders, rewards)<br>Motivation (the presence of mental health condition)<br>Personal traits (reducing embarrassment about mental health problem, self-reliance)
BevanJones 2020	Young people	Perceived unhelpfulness (information already known and not specific)<br>Lack of fit (not relevant to those not experiencing depression difficulties)<br>Unfavorable program design (too much textual)<br>Concern about the safety (unnecessarily worry)	Ease of use<br>Perceived usefulness (comprehensive information)<br>Perceived fit (individualization)<br>Connectedness (support)<br>Program design (wording, user-friendly and attractive design, using illustrations and animations, clear structure, favorable feature: app, reminder, rewards)<br>Perceived need (the presence or being at risk of mental health problems)<br>Perceived motivation (finding W-MHIs acceptable)<br>Feeling normal (overcoming stigma)
	Professionals	Technical factors (keeping program up to date)	Young people-related factors (interest)<br>Intervention-related factors (perceived usefulness for young people, program layout, information)
	Parents (as part of the program)		Ease of use<br>Perceived usefulness (helpful, adequate information)<br>Program design (engaging, friendly illustrations and animations without being patronizing, "holistic" and "systematic" approach)
Bohleber 2016	Young people	Lack of time (20%)<br>Technical factors (forgetting log-in details, 8.3%)<br>Perceived unhelpfulness (not seeing the benefit, 23.3%)<br>Low perceived need (lack of mental health services, disinterest)<br>Social factors (few active users, 5%)	Perceived usefulness (more specific information compared to apps)<br>Connectedness (social support, 10-23.3%; peer mentoring)<br>Program design (attractive design, clear structure)<br>Favorable features (psychological test, 6.7%)<br>Perceived motivation (enjoyment, 21.7%)
Bowman 2020	Young LGBT	Technical problems (limited Internet access)<br>Online network (impersonal nature)<br>Low perceived need (lack of mental health services, scattered information)<br>Uncertain about its effectiveness<br>Stigma	Ease of use<br>Online network (impersonal nature-prefer to talk online)<br>Program design (informal communication)<br>Positive attitude (finding W-MHIs acceptable)

Author, year	Participants	Barriers	Facilitators
	Service providers	Practical factors (overlapping services)<br>Logistical factors (mental health funding)<br>Intervention-related factor (hard to navigate)	Intervention-related factors (anonymity and privacy for users, connectedness)
Bradley 2012	Young people	Technical problems (problem loading program)<br>Lack of fit (too long modules, difficulty of tasks)<br>Unfavorable program design (black and white design)<br>Low perceived need (lack of motivation, lack of mental health knowledge)<br>Concern about privacy and confidentiality	Convenience (anytime, easy to incorporate into schedule)<br>Ease of use<br>Perceived usefulness (learning practical techniques and skills, gaining different perspectives of the problems)<br>Perceived fit (relatedness)<br>Program design (user-friendly design, easy to navigate, pictures)<br>Perceived motivation<br>Trust (privacy and confidentiality)<br>Feeling of safety (non-judgment)
Bunnell 2017	Young people	Busyness (72.4-75.9%)<br>Perceived unhelpfulness (15.5-21.2%)<br>Concern about privacy and confidentiality (3.7-10.6%)	
	Families (as part of the program)	Too busy<br>Required Internet access<br>Perceived unhelpfulness<br>Irrelevance<br>Concern about privacy and security	
Calear 2013	Young people		Sociodemographic (younger age, location)<br>Perceived need (the presence of mental health condition)<br>Personal traits (self-esteem)
Chan 2016	Young people	Perceived unhelpfulness (hard to portray feelings in writing)<br>Low perceived need (lack of familiarity with mental health services - scattered information)<br>Uncertainty about the effectiveness and credibility, concern about privacy and confidentiality<br>Fear of problem being worsened	Convenience (anytime)<br>Connectedness (connect with others)<br>Feeling normal (overcoming stigma)
Clark 2018	Young people	Lack of fit (lack of autonomy)<br>Online network (impersonal nature)<br>Low perceived need (lack of mental health knowledge)<br>Concern about privacy and confidentiality	Ease of use<br>Perceived usefulness (immediate benefits)<br>Connectedness (impersonal nature)<br>Program design (wording, informal communication)<br>Perceived need (severity of mental health symptoms)<br>Trust (privacy and confidentiality)<br>Feeling of safety (non-judgment)

Author, year	Participants	Barriers	Facilitators
Dai 2022	Young people		Ease of use<br>Perceived usefulness (helpful content)<br>Positive attitude
Dobias 2022	Young people	Perceived unhelpfulness<br>Online network (too simplistic, impersonal nature, lack of connection)<br>Low perceived motivation (hard to engage due to the presence of symptom)<br>Concern about short-term effectiveness	Perceived usefulness (improving symptoms, raising mental health awareness)
Ellis 2013	Young men		Perceived fit (relatedness)<br>Program design (favorable contents, e.g., information/multimedia content, questions, and answers via SMS/email, 28.8-48.1%)<br>Trust about its credibility, belief about privacy and confidentiality<br>Sociodemographic (age)
Geirhos 2022	Young people	Lack of fit (inappropriate length, 13%)<br>No motivation or changes in life circumstances<br>Required too much time or effort	Perceived usefulness<br>Perceived fit (appropriate length, 87%)
Gericke 2021	Young people	Lack of time (busyness)<br>Required effort<br>Online network/Limited support (long delay of support, lack of human contact)<br>Unfavorable program design (textual)<br>Lack of motivation	Convenience (anytime)<br>Perceived usefulness (learning practical techniques and skills, self-expression and understanding themselves, relieving symptoms)<br>Perceived fit (relatedness, individualization, autonomy)<br>Program design (initial screening test)<br>Perceived motivation (finding W-MHIs acceptable)<br>Privacy and confidentiality<br>Feeling normal (overcoming stigma)
González-García 2021	Young people	Perceived unhelpfulness (1.51%)<br>Lack of fit (difficult tasks, 85.71%)<br>Feeling insecure about meditation (72.37%)<br>Negative attitude (self-criticism, 56.58%)	Perceived usefulness (87.7%)
Hämäläinen 2021	Young people		Sociodemographic (female)<br>Personal traits (self-regulation)
Iloabachie 2011	Young people		Perceived usefulness (learning practical techniques and skills)<br>Perceived fit (individualization)<br>Connectedness (connect with others)<br>Program design (post message)

Author, year	Participants	Barriers	Facilitators
	Parents (as part of the program)	Content (presenting depression as an illness was negative)<br>Lack of interactivity	Perceived usefulness for parents and young people
Kahl 2020	Young people		Convenience (any time, 87.5%)<br>Perceived usefulness (learn new things, self-reflection, 69.2-72.9%)<br>Perceived fit (relevance: 83.1%)
Kanuri 2020	Young people	Technical factors<br>Layout (web-app design)<br>Lack of fit (length of videos)<br>Unfavorable program design (weblink to third party, complex and inconsistent structure)<br>Low perceived need (lack of mental health knowledge)<br>Concern about its privacy and confidentiality<br>Stigma<br>Personal trait (neutrally confident to use)	Ease of use<br>Perceived usefulness (immediate benefits)<br>Perceived fit (relatedness, 10-15mins per module)<br>Program design (well-integrated)<br>Trust about its effectiveness
Karim 2021	Young people	Lack of time (busyness)<br>Program design (lack of interactivity)<br>Concern about privacy and confidentiality	Perceived usefulness* (gaining different perspectives of the problems, self-expression)<br>Connectedness (connect with others, gaining support)<br>Layout (presentation, design)<br>Perceived motivation (interest)<br>Trust (privacy and confidentiality)
Karyotaki 2022	Young people	Lack of time<br>Technical problems (no internet access)<br>Low perceived need (need different help)<br>Lack of motivation (lack of enjoyment)	
Kurki 2018	Nurses	Logistical factors (increased workload, limited resources)<br>Provider-related factors (resistance to change, concern about privacy for users)	Practical factors (flexibility)<br>Logistical factors (organizational support)<br>Young people-related factors (positive attitude about W-MHIs)
Lattie 2017	Young people	Technical problems (problem loading program)<br>Lack of fit (lack of individualization)<br>Lack of support (insufficient guide for support)<br>Unfavorable program design (lack of interactivity, peer group)	Perceived usefulness (learning practical techniques and skills)<br>Program design (ability to track progress; language: easy to understand)<br>Perceived fit (relevance)<br>Connectedness (instant support)<br>Perceived enjoyment

Author, year	Participants	Barriers	Facilitators
Leech 2020	Young people	Perceived unhelpfulness (38%)	Perceived usefulness (30%)<br>Connectedness (gaining support, 92%)<br>Trust (treatment credibility, privacy and confidentiality, 87-96%)<br>Sociodemographic (male: seeking informal support online, living with direct family: access mental health via social media)
Lenhard 2016	Young people	Lack of motivation (preference towards face-to-face sessions)	Convenience (anytime, easy to incorporate into schedule)<br>Perceived usefulness (improving mental knowledge, self-expression)<br>Perceived fit (relatedness, autonomy)<br>Connectedness (instant support)<br>Perceived motivation<br>Trust (belief about its effectiveness, building trust)<br>Feeling normal<br>Feeling of safety (feeling secured)
Lilja 2021	Young people	Lack of time (busyness)<br>Personal trait (lack of confidence)<br>Uncertain about its effectiveness	Convenience (anytime)<br>Perceived usefulness (improving mental health knowledge)<br>Perceived fit (autonomy, individualization)<br>Program design (combined text, pictures and films)
	Parents	Doubt about program effectiveness<br>Limited insights into treatment (their role)	Perceived usefulness for young people
Lillevoll 2014	Young people	Lack of time (busyness, 57.7%)<br>Low perceived need (prefer to talk to someone, 28.9%)<br>Uncertain about treatment efficacy, concern about privacy and confidentiality (no computer access where they can work undisturbed, 14.1%)	Social influence (social norms)<br>Sociodemographic (educational level)

Author, year	Participants	Barriers	Facilitators
Lindegaard 2022	Young people	Lack of time<br>Required concentration and effort to understand<br>Technical problem (struggling with setting up password)<br>Lack of fit (cultural difference and irrelevant content)<br>Online network (preferred video call/conversation to with therapists instead of text messaging, lack of human contact and support)<br>Low motivation (the presence of mental illness symptom; hard to use for those with severe condition)<br>Low trust in program effectiveness<br>Personal trait (lack of confidence)	Ease of use<br>Perceived usefulness (helpful content)<br>Program design (text: easy to understand and logical structured)<br>Perceived fit (culturally appropriate)<br>Ability to reach more people (due to the convenience)<br>Trust (anonymity)
Mamdouh 2022	Young people	Cost<br>Technical difficulties<br>Concern about the program (e.g., validity and reliability, confidentiality, and privacy)<br>Low perceived need (uncertainty toward sand unfamiliarity with W-MHIs)	Cost-effective<br>Convenience<br>Connectedness (rapid response)<br>Program design (user-friendly)<br>Trust (privacy, anonymity, trustworthy national web-based platform)<br>Decreased stigma<br>Sociodemographic (gender, location, e.g., males and individuals living in urban areas knew more about existing mental health websites and apps)
Manicavasagar 2014	Young people	Lack of time (busyness)<br>Technical problems (limited Internet access)<br>Perceived unhelpfulness (repetitive information)<br>Lack of fit (age-inappropriateness, unrelatedness)<br>Unfavorable program design (textual, lack of interactivity, use of emails, preference for smartphone access)	Ease of use (90%)<br>Perceived usefulness (improving mental health knowledge, understanding themselves)<br>Perceived motivation (enjoyment, 79-84%)
Mar 2014	Young people	Unfavorable program design (textual, dark imagery, lack of simplicity)<br>Low perceived need<br>Concern about building trust online<br>Concern about the safety (problem worsened)	Perceived usefulness (improving mental health knowledge)<br>Connectedness (ability to connect with others, instant support, impersonal nature)<br>Program design (user-friendly, simple, goal setting feature)<br>Trust about privacy and confidentiality

Author, year	Participants	Barriers	Facilitators
Marko 2010	Young people		Convenience (any time, 59%)<br>Ease of use (62.83%)<br>Perceived fit (easy task, 42%)<br>Trust (belief about treatment efficacy, 65-70%)<br>Sociodemographic (gender: male had lower intention to use)
Mawdsley 2022	Young people	Nature of online network (struggle to communicate thoughts and feelings via chat, impersonal nature, i.e., feel disconnected, inhibit the establishment of therapeutic relationship)<br>Concern about the program and therapist (pre-existing anxieties: fear of being rejected by counselors; concern that they might not be able to help them)	Perceived usefulness (help them to reach their goals/outcome, positive sensations after the counselling)<br>Connectedness (impersonal nature)<br>Perceived motivation<br>Trust (optimism about the program can help them)<br>Trust/belief (privacy, confidentiality)
	Counsellors	Intervention-related factors (facelessness causing ambiguity in communication)<br>Young people -related factors (lack of motivation or low mood, low perceived rapport with counsellors, the presence of health condition, e.g., autism, medication, dyslexia)	Perceived usefulness for young people<br>Intervention-related factor (faceless contact)<br>Young people -related factors (motivation)
McDanal 2022	Young people	Lack of fit (inappropriate length, applicable to only a certain age range)<br>Unfavorable program design (difficult to navigate, lack of interactivity)<br>Lack of enjoyment	Ease of use<br>Perceived usefulness (improving mood, providing applicable skills or insight)<br>Perceived fit (content - inclusive about genders and sexuality)<br>Connectedness (feeling less isolated or alone)
Nicolaou 2022	Young people	Technical difficulties<br>Perceived unhelpfulness (the repetition of questionnaires)<br>Lack of fit (length of the program 29% - required time, number of sessions, lack of autonomy and flexibility, i.e., inability to pause the sessions, requiring participants to complete each session in its entirety at once)<br>Unfavorable features	Low cost (free services, 16.1%)<br>Convenience (no need to go anywhere, continuous support whenever needed, 6.5%)<br>Perceived usefulness (learning something new, 92%, overall helpfulness, 82%, helping deal with body-related thoughts, 48-52%)<br>Perceived fit (appropriate duration, 45%)<br>Connectedness (getting expert advice, 54.8%)<br>Perceived motivation (curiosity, 22.6%)<br>Trust/belief (anonymity, 19.4%)

Author, year	Participants	Barriers	Facilitators
O'Bree 2021	Young people	Lack of time (busyness)<br>Lack of fit (age-inappropriateness, lack of individualization)<br>Online network (support below expectation)<br>Unfavorable program design (not user-friendly)<br>Lack of motivation (disinterest)	Convenience (anywhere)<br>Perceived usefulness (learning practical techniques and skills, immediate benefits)<br>Perceived fit (relatedness)<br>Connectedness (connect with others, receiving support)<br>Program design (bespoke therapy comics)<br>Perceived motivation (finding W-MHIs acceptable)<br>Feeling normal<br>Feeling of safety (non-judgment)
Păsărelu 2021	Young people		Perceived usefulness<br>Program design (user-friendly)<br>Perceived enjoyment
Pine 2020	Teachers and health professionals	Provider-related factors (doubt about effectiveness, concern about lack of face-to-face contact)	Practical factors (flexibility, easy to access)<br>Provider-related factors (positive attitude about W-MHIs)
Pretorius 2010	Young people	Lack of flexibility (time inflexibility)<br>Technical problems (loading program)<br>Perceived unhelpfulness (repetitive information)<br>Lack of fit (difficulty of tasks)<br>Online network (long delay of support, impersonal nature)<br>Unfavorable program design (overusing pictures, disliked audio voice)<br>Low perceived need (lack of mental health knowledge, lack of motivation)<br>Uncertainty about program effectiveness<br>Concern about the safety (problem worsened)<br>Social factors (few active users)	Convenience (working at own pace, anywhere)<br>Ease of use<br>Perceived usefulness (improving mental health knowledge)<br>Perceived fit (individualization)<br>Connectedness (connect with others, persistent support)<br>Program design (well presented, favorable features, i.e., message boards, ability to track progress)<br>Perceived need and motivation (past unhelpful experience with face-to-face therapies, finding W-MHIs acceptable)<br>Trust (privacy and confidentiality)<br>Feeling normal
Price 2015	Young people	Lack of time (busyness, 73.61%)<br>Concern about privacy and confidentiality (3.8-8%)	Ease of use (74.3%)<br>Sociodemographic (gender, family status)<br>Perceived need (male having lower intention to use, presence of mental health condition)

Author, year	Participants	Barriers	Facilitators
Punukollu 2020	Young people	Lack of time (busyness)<br>Technical problems<br>Perceived unhelpfulness (information already known)<br>Online network/Limited support (insufficient guide)<br>Unfavorable program design (wording: difficult to understand, little continuity across tutorials, lack of interactivity)<br>Low perceived need	Perceived usefulness (improving mental health knowledge, learning practical techniques and skills)<br>Feeling normal
	Teachers	Intervention-related factor (too much tutorial content, lack of support/information regarding sensitive topic)	Intervention-related factor (helpful support material; perceived usefulness for pupils)
Richiello 2022	Counsellors	Technical issues (slow Internet connection, suddenly being logged out and sudden interruption of the conversation)<br>Logistical factor (lack of prompt support in out-of-business hours, lack of training)<br>Intervention-related factor (impersonality, e.g. hard to reach relational depth with clients, duration of session, e.g. writing takes longer impact quality of the session)<br>Provider-related factor (less experienced with technology, not confident to deliver webchat; doubts about its effectiveness)	Practical factor (working remotely, timetable flexibility)<br>Logistic factor (organizational support during the working hours, providing training)<br>Provider-related factor (familiar with technology + training -> Confidence (perform the behavior)<br>Intervention-related factor (impersonal nature, perceived usefulness for young people, connectedness)
Rickwood 2019	Young people		Connectedness (availability and level of support)<br>Perceived fit (modules >30mins)<br>Perceived need (not previously access center)
Sansom-Daly 2019	Young people	Lack of time (busyness)<br>Technical factors (71% sessions)	Perceived usefulness (improving mental health knowledge, understanding themselves, 61-70%)<br>Feeling normal<br>Sociodemographic (younger age)
	Caregivers		Perceived usefulness (11-25%)<br>Low perceived burden (100%)

Author, year	Participants	Barriers	Facilitators
Santesteban-Echarri 2017	Young people	Perceived unhelpfulness (information already known)<br>Lack of fit (inappropriate moderator's approach, lack of autonomy, too long modules)<br>Online network (support below expectation)<br>Unfavorable program design (textual, overwhelming information and not well organized, clunky activities)<br>Personal trait (shyness)<br>Low perceived need and motivation (disinterest)<br>Social factors (few active users, not knowing other users)	Perceived usefulness (learning practical techniques and skills, improving mood)<br>Perceived fit (relatedness, individualization)<br>Connectedness (connect with others, instant support, impersonal nature)<br>Program design (attractive design, clear structure, interactivity)<br>Perceived motivation (interest, mental health condition)<br>Trust (privacy and confidentiality)<br>Feeling normal<br>Feeling of safety (non-judgment)
Sawrikar 2022	Young people		Perceived usefulness<br>Trust
Schleider 2020	Young people		Ease of use<br>Perceived usefulness (message content)<br>Connectedness (help their peers)<br>Program design (language: easy to understand)<br>Perceived motivation (enjoyment)<br>Sociodemographic (age)
Schmitt 2022	Young people		Perceived ease of use<br>Convenience (easy to include in the daily routine)<br>Perceived usefulness (effective, learn coping skills)<br>Program design (easy to understand, videos, avatar, homework)<br>Perceived enjoyment<br>Connectedness (therapists: helpful, respect each other, caring)
	Parents (as part of the program)		Perceived usefulness of parent's section was useful<br>Views about their children's experience with the platform: same as adolescents view, except the inclusion in daily routine M7.87
Shandley 2010	Young people	Technical problems (required multiple downloads)	Ease of use (90%)<br>Sociodemographic (gender: female having higher usage)<br>Perceived enjoyment (interesting and fun)

Author, year	Participants	Barriers	Facilitators
Smart 2021	Young people	Required effort<br>Technical problems (connection issues)<br>Perceived unhelpfulness (information already known, repetitive information)<br>Lack of fit (age-inappropriateness, unrelatedness, lack of flexibility in format)<br>Online network (impersonal nature - not same experience as face-to-face therapies, limited support availability)<br>Unfavorable program design (textual)<br>Personal trait (shyness)<br>Lack of motivation	Convenience (anytime)<br>Perceived usefulness (increasing mental health knowledge, self-expression)<br>Perceived fit (age appropriate, reasonable length of module)<br>Connectedness (instant support)<br>Program design (wording: informal, feature: quizzes, rewards)<br>Feeling normal
Sobowale 2016	Young people	Lack of fit (age-inappropriateness, length of modules-reluctant to spend >30mins)<br>Low perceived need (lack of mental health knowledge)<br>Concern about privacy and confidentiality	Cost (low cost)<br>Ease of use<br>Perceived usefulness (increasing mental health knowledge)<br>Perceived motivation (finding W-MHIs acceptable)<br>Social influence
	Parents	Required Internet access<br>Perceived risk of internet addiction	Perceived usefulness for young people<br>Online network (young people can exchange information comfortably)<br>Trust about program (credibility)
Sweeney 2016	Young people	Lack of time (busyness) (42.4%)<br>Technical factors (38.5-39%)<br>Perceived unhelpfulness (too general information: 53.8%)<br>Lack of fit (difficult tasks, 37.5%)<br>Online network (lack of support, 51.5-61.5%)<br>Lack of motivation (little interest, 45.7%)<br>Concern about privacy and confidentiality (51.5%)	Cost (low cost, 76.4%)<br>Convenience (anywhere, anytime, 62.5-79.9%)<br>Easy to use (72.1%)<br>Perceived usefulness (83.3%)<br>Program design (feature: tracking progress, interactivity, 61.1-72.1%)<br>Personal trait (reducing embarrassment)<br>Perceived enjoyment (interesting, preferred over face-to-face programs 33.3-37.5%)

Author, year	Participants	Barriers	Facilitators
vanDalen 2022	Young people	Busyness/forget<br>Technical factors (website did not work properly with 2-factor authentication)<br>Lack of fit (session was too long, too childish for those >15years, cannot tailor to individual needs; lack of flexibility: journal just available at some point in the session)<br>Low perceived need (prefer to talk to someone directly)<br>Unfavorable program design (preferred audios, more videos, shorten texts)<br>Social factors (no activities on the forum)	Convenience (do in own time when convenient)<br>Perceived ease of access<br>Perceived usefulness (learning about the think- feel-do technique was very helpful, Change the way I think: better understanding of other people's thoughts and intentions; change in her self-esteem)<br>Perceived fit (relatable, session length was appropriate, suit for adolescents 12-18years)<br>Program design and features (clear explanation, activities and quiz : helped embed key learning, Structure: logical; colorful website, reminder helpful)<br>Perceived motivation (curious)<br>Trust/belief (anonymity; not having to disclose personal information)
Watkins 2017	Black men	Lack of fit (inappropriate language)	Convenience (around internet platform)<br>Perceived usefulness (increasing mental health knowledge, learning practical techniques and skills, self-expression, immediate benefits)<br>Connectedness (connect with others, support)<br>Favorable features (questions and prompts)<br>Trust (privacy and confidentiality)<br>Feeling of safety (non-judgment)
Weineland 2020	Primary care therapists	Practical factors (concern about the accessibility and flexibility)<br>Provider-related factors (lack of trust in information technology)<br>Young people-related factors (health condition, demanding responsibility)<br>Intervention-related factors (inappropriate regarding age and content, standardized structure, online communication conveying less information)	Practical factors (flexibility, easy to use)<br>Logistical factors (increased task variety)<br>Provider-related factors (belief about treatment result)<br>Young people-related factors (facing difficulty with seeing psychologists, the role of therapists)<br>Intervention-related factors (perceived usefulness for young people and providers, program layout, pre-determined structure, individualization via messaging)
Wetterlin 2014	Young people	Lack of awareness of mental health services (5.8% access website previously)	Connectedness (connect with others, 74.8-83.9%)<br>Program design (mainly text, using videos/pictures, favorable features, i.e., quizzes, resource lists, etc., 82.9%)<br>Trust (treatment credibility, privacy and confidentiality, 87.7-88.3%)

Author, year	Participants	Barriers	Facilitators
Windler 2019	Young people	Lack of fit (moderators' approach, e.g., self-disclosure: commenting on their own experience)	Perceived fit (moderator's approach, e.g., providing emotional support, sharing expertise)<br>Connectedness (receiving support)
	Moderators	Technical problem (iterative updates to website functionality)<br>Young people-related factor ('a unique type of adolescent want to engage', social factor: limited users)	Logistical factor (providing training)<br>Provider-related factor (perception about their role to keep the site and users safe)
Woolderink 2015	Young people	Perceived unhelpfulness (repetitive information)<br>Lack of fit (age-inappropriateness)<br>Online network (below expectation)<br>Technical problems (problem loading program, limited Internet access)<br>Concern about privacy and confidentiality	Ease of access<br>Perceived usefulness (increasing mental health knowledge, learning practical techniques and skills)<br>Perceived fit (age appropriate, autonomy)<br>Connectedness (connect with others, easy to access the support)<br>Program design (attractive design, favorable features, i.e., rating your week)<br>Perceived motivation<br>Trust (privacy and confidentiality)<br>Feeling of safety (feeling secured)<br>Social influence
	Providers	Practical factor (technical problem)<br>Logistical factors (limited resources)	Practical factors (flexibility)<br>Intervention-related factors (anonymity for users, age-appropriateness, clear website and chat box, important content was covered)
Wuthrich 2021	Young people	Online network (limited contact with therapists- prefer more frequent contact)	Convenience (self-paced)
	Clinicians	Practical factor (lack of flexibility: required users to complete internet-based CBT before face-to-face therapy, scheduled phone calls were time-consuming)<br>Logistical factor (work allocation being not set up, perception about their role)<br>Intervention-related factor (structured internet-based CBT causing clinician bored, hard to build rapport with clients over the telephone)<br>Provider's concern about its effectiveness (not appropriate for severe cases, not sufficient to address the underlying causes of anxiety, not lasting therapeutic change)<br>Young people-related factor (preference to face-to-face services)	Increase reach (rural and remote adolescents and those in the waiting list can reach W-MHIs)<br>Logistic factor (useful training)<br>Intervention-related factor (perceived usefulness for providers, e.g., potential aiding staff development, and for users)

Author, year	Participants	Barriers	Facilitators
Zeiler 2021	Young people	Lack of time (busyness)<br>Technical problems (limited Internet access)<br>Lack of fit (difficulty of tasks)<br>Online network (impersonal nature)<br>Personal trait (lack of determination)<br>Uncertain about its effectiveness<br>Concern about privacy and confidentiality<br>Stigma	Low or no cost<br>Convenience (anytime, easy to incorporate into YP's schedule)<br>Ease of access<br>Perceived usefulness (increasing mental health knowledge)<br>Perceived fit (autonomy)<br>Connectedness (impersonal nature)<br>Layout (wording, attractive and responsive design, presentation)<br>Favorable features (interactivity)<br>Perceived motivation (finding W-MHIs acceptable)<br>Trust (program credibility, privacy, and confidentiality)<br>Feeling normal (overcoming stigma)
	Stakeholders (teachers, school psychologists and policy makers)	Practical factors (lack of time, technical problem - Internet is a must)<br>Logistical factors (limited resources)<br>Provider-related factors (lack of knowledge about online programs, doubt about effectiveness and privacy for users)<br>Intervention-related factors (perceived risk to young people, lack of face-to-face contact)	Practical factors (flexibility: anytime, anywhere, easy to integrate into school curriculum, easy to use)<br>Provider-related factor (acceptance of W-MHI)<br>Young people-related factors (low cost, mental health condition, facing difficulty with seeing psychologists)<br>Intervention-related factors (anonymity for users, perceived usefulness for young people and providers, connectedness)
