# Supplementary material for: Barriers to and facilitators of user engagement with web-based mental health interventions in young people: a systematic review
Source: Eur Child Adolesc Psychiatry. 2024 Feb 14;34(1):83–100. doi: 10.1007/s00787-024-02386-x (PMC11805866; doi:10.1007/s00787-024-02386-x)
Supplement: Supplementary file 5 — Supplementary file5 (PDF 217 KB) [file 787_2024_2386_MOESM5_ESM.pdf]

Journal: European Child and Adolescent Psychiatry

Article title: “Barriers to and facilitators of user engagement with web-based mental health intervention in young people: a systematic review”

Authors:
Thi Quynh Anh Ho; Long Khanh-Dao Le; Lidia Engel; Ngoc Le; Glenn Melvin; Ha N.D. Le*; Cathrine Mihalopoulos*

*: Joint senior authors

Corresponding author:
Thi Quynh Anh Ho – School of Health and Social Development, Deakin University, Melbourne, Victoria, Australia tqho@deakin.edu.au

Supplementary material 5 – Quality assessment

First author, year	1. Qualitative studies					2. Quantitative studies – randomized controlled trial					3. Quantitative studies – non-randomized					4. Quantitative descriptive studies					5. Mixed methods studies					Score	Rating
	1.1	1.2	1.3	1.4	1.5	2.1	2.2	2.3	2.4	2.5	3.1	3.2	3.3	3.4	3.5	4.1	4.2	4.3	4.4	4.5	5.1	5.2	5.3	5.4	5.5		
Anttila 2019											Y	Y	Y	N	Y											4	High
Anttila 2020																					Y	Y	Y	Y	N	4	High
Aspvall 2020																Y	N	Y	Y	Y						4	High
Babiano-Espinosa 2021																Y	N	Y	Y	Y						4	High
Bailey 2021	Y	Y	Y	Y	Y																					5	High
Bannink 2014						Y	Y	Y	N	Y																4	High
Banwell 2022																Y	Y	Y	Y	Y						5	High
Bautista 2022						Y	Y	Y	N	Y																4	High
Beames 2021																Y	N	Y	-	Y						3	Moderate
Benjet 2020																Y	N	Y	Y	Y						4	High
BevanJones 2020																					N	Y	N	Y	Y	3	Moderate
Bohleber 2016																					N	Y	N	-	N	1	Low
Bowman 2020	Y	Y	Y	Y	Y																					5	High
Bradley 2012	Y	Y	Y	Y	Y																					5	High
Bunnell 2017																Y	Y	Y	Y	Y						5	High
Calear 2013						Y	N	Y	N	Y																3	Moderate
Chan 2016	Y	Y	Y	Y	Y																					5	High
Clark 2018	Y	Y	Y	Y	Y																					5	High

First author, year	1. Qualitative studies					2. Quantitative studies – randomized controlled trial					3. Quantitative studies – non-randomized					4. Quantitative descriptive studies					5. Mixed methods studies					Score	Rating
	1.1	1.2	1.3	1.4	1.5	2.1	2.2	2.3	2.4	2.5	3.1	3.2	3.3	3.4	3.5	4.1	4.2	4.3	4.4	4.5	5.1	5.2	5.3	5.4	5.5		
Dai 2022						Y	Y	Y	N	Y																4	High
Dobias 2022																Y	N	Y	Y	Y						4	High
Ellis 2013																					Y	Y	Y	Y	Y	5	High
Geirhos 2022						Y	N	Y	N	Y																3	Moderate
Gericke 2021	Y	Y	Y	Y	Y																					5	High
González-García 2021																Y	N	Y	Y	Y						4	High
Hämäläinen 2021																Y	Y	N	Y	Y						4	High
Iloabachie 2011																					Y	Y	Y	Y	N	4	High
Kahl 2020																Y	N	N	Y	Y						3	Moderate
Kanuri 2020																					N	Y	Y	Y	N	3	Moderate
Karim 2021																					N	Y	Y	Y	N	3	Moderate
Karyotaki 2022						Y	N	N	N	Y																2	Low
Kurki 2018	Y	Y	Y	Y	Y																					5	High
Lattie 2017																					N	Y	Y	Y	Y	4	High
Leech 2020																Y	N	Y	-	Y						3	Moderate
Lenhard 2016	Y	Y	Y	Y	Y																					5	High
Lilja 2021																					Y	Y	Y	Y	Y	5	High
Lillevoll 2014						Y	Y	N	N	N																2	Low
Lindegaard 2022																					N	Y	Y	Y	N	3	Moderate
Mamdouh 2022																N	N	Y	Y	Y						3	Moderate
Manicavasagar 2014																					N	Y	Y	Y	N	3	Moderate
Mar 2014	Y	N	Y	Y	Y																					4	High
Marko 2010																Y	N	Y	Y	Y						4	High
Mawdsley 2022	Y	Y	Y	Y	Y																					5	High
McDanal 2022																					N	Y	Y	Y	N	3	Moderate
Nicolaou 2022																N	Y	Y	N	Y						3	Moderate
O'Bree 2021	Y	Y	Y	Y	Y																					5	High
Păsărelu 2021																Y	N	Y	Y	Y						4	High
Pine 2020	Y	Y	Y	Y	Y																					5	High

First author, year	1. Qualitative studies					2. Quantitative studies – randomized controlled trial					3. Quantitative studies – non-randomized					4. Quantitative descriptive studies					5. Mixed methods studies					Score	Rating
	1.1	1.2	1.3	1.4	1.5	2.1	2.2	2.3	2.4	2.5	3.1	3.2	3.3	3.4	3.5	4.1	4.2	4.3	4.4	4.5	5.1	5.2	5.3	5.4	5.5		
Pretorius 2010	Y	Y	Y	Y	Y																					5	High
Price 2015																Y	Y	N	-	Y						3	Moderate
Punukollu 2020	Y	Y	Y	Y	Y																					5	High
Richiello 2022	Y	Y	Y	Y	Y																					5	High
Rickwood 2019																Y	Y	N	N	Y						3	Moderate
Sansom-Daly 2019																					N	Y	Y	Y	N	3	Moderate
Santesteban-Echarri 2017	Y	Y	Y	Y	Y																					5	High
Sawrikar 2022																N	N	Y	Y	Y						3	Moderate
Schleider 2020																Y	Y	Y	Y	Y						5	High
Schmitt 2022																Y	N	Y	Y	Y						4	High
Shandley 2010																Y	N	N	-	Y						2	Low
Smart 2021	Y	Y	Y	Y	Y																					5	High
Sobowale 2016	Y	Y	Y	Y	Y																					5	High
Sweeney 2016																Y	N	N	Y	N						2	Low
vanDalen 2022																					N	N	Y	Y	N	2	Low
Watkins 2017	Y	Y	Y	Y	Y																					5	High
Weineland 2020	Y	Y	Y	Y	Y																					5	High
Wetterlin 2014																Y	N	Y	Y	-						3	Moderate
Windler 2019	Y	Y	Y	Y	Y																					5	High
Woolderink 2015	Y	Y	Y	Y	Y																					5	High
Wuthrich 2021																					N	Y	Y	Y	N	3	Moderate
Zeiler 2021																					Y	Y	N	Y	Y	4	High
